# Comparative plastome genomics, taxonomic delimitation and evolutionary divergences of *Tetraena hamiensis* var. *qatarensis* and *Tetraena simplex* (Zygophyllaceae)

**DOI:** 10.1038/s41598-023-34477-1

**Published:** 2023-05-08

**Authors:** Waqar Ahmad, Sajjad Asaf, Ahmed Al-Rawahi, Ahmed Al-Harrasi, Abdul Latif Khan

**Affiliations:** 1 Natural and Medical Sciences Research Centre, University of Nizwa, Nizwa, 616 Oman; 2 Department of Engineering Technology, University of Houston, Sugar Land, TX 77479 USA; 3 Department of Biology and Biochemistry, University of Houston, Houston, USA

**Keywords:** Genomics, Plant biotechnology, Sequencing

## Abstract

The *Zygophyllum* and *Tetraena* genera are intriguingly important ecologically and medicinally. Based on morphological characteristics, *T. hamiensis* var. *qatarensis,* and *T. simplex* were transferred from *Zygophyllum* to *Tetraena* with the least genomic datasets available. Hence, we sequenced the *T. hamiensis* and *T. simplex* and performed in-depth comparative genomics, phylogenetic analysis, and estimated time divergences. The complete plastomes ranged between 106,720 and 106,446 bp—typically smaller than angiosperms plastomes. The plastome circular genomes are divided into large single-copy regions (~ 80,964 bp), small single-copy regions (~ 17,416 bp), and two inverted repeats regions (~ 4170 bp) in both *Tetraena* species. An unusual shrinkage of IR regions 16–24 kb was identified. This resulted in the loss of 16 genes, including 11 *ndh* genes which encode the NADH dehydrogenase subunits, and a significant size reduction of *Tetraena* plastomes compared to other angiosperms. The inter-species variations and similarities were identified using genome-wide comparisons. Phylogenetic trees generated by analyzing the whole plastomes, protein-coding genes, *matK*, *rbcL*, and *cssA* genes exhibited identical topologies, indicating that both species are sisters to the genus *Tetraena* and may not belong to *Zygophyllum*. Similarly, based on the entire plastome and proteins coding genes datasets, the time divergence of *Zygophyllum* and *Tetraena* was 36.6 Ma and 34.4 Ma, respectively. *Tetraena* stem ages were 31.7 and 18.2 Ma based on full plastome and protein-coding genes. The current study presents the plastome as a distinguishing and identification feature among the closely related *Tetraena* and *Zygophyllum* species. It can be potentially used as a universal super-barcode for identifying plants.

## Introduction

Accurately identifying plant species is necessary for the long-term use and conservation of biological resources^1,2^. Traditional taxonomy is focused on morphological characteristics, requiring taxonomists with extensive taxonomy knowledge and careful examination of specimens^3^. However, when there are many specimens in a study^4^, morphological analysis can be time-consuming, leading to a significant decrease in the authenticity of species identification. Furthermore, if available specimens belong to a complex genus, accurate identification using the standard method may be inefficient^1,5^. Because of the limited and uncertain diagnostic attributes, the morphology-based process of identifying plant species has yet to be discovered. Molecular markers are very useful in plant identification and have played a pivotal role in systematics for a long time. However, the currently available loci (DNA) only work for the closely related species, not all^6^.

Short, standardized DNA segments are used in DNA barcoding as an additional tool for morphological taxonomy. Paul Hebert first suggested it in 2003, and it was soon recognized as a valuable method for identifying and discovering new species^7^. Multiple genes, such as three plastid regions (e.g., *rbcL, matK*, and *trnH- psbA*) and nuclear DNA (Internal Transcribe Spacer—ITS) are usually recognized as universal DNA barcodes for terrestrial plants. However, only some barcodes can successfully identify most species^8,9^. DNA barcodes may be ineffective for complex taxonomic groups, especially in newly evolved and rapidly expanded taxa, for subspecies discrimination^10^. Therefore, there is a critical need to create new, reliable methods to meet the requirements of classifying complicated plant species. With the current advancement in next-generation sequencing (NGS) technologies, the accuracy and quality of DNA sequencing have considerably improved the species discrimination for complex genera^11,12^.

The Chloroplast genome (plastome) is an excellent resource for resolving the tree of life and identifying taxa^11,13^ Numerous studies have demonstrated that the chloroplast genome is a robust and effective method for uncovering plant phylogeny and evolutionary history more accurately^14,15^. Unlike the four standard barcodes, due to their significant genetic variety, DNA super-barcode, which employs the entire chloroplast genome sequence, can distinguish between closely related species^11,16,17^. Despite this, DNA super-barcodes face criticism and challenges in species identification, such as high sequencing costs, lack of a comprehensive cp-genome database, and difficulty tracking species boundaries^18^. In recent years, as the cost of NGS sequencing has decreased and analysis methods for genomes have improved, large plastomes datasets have become available in GenBank. This leads to several genera having corresponding reference genomes for species identification and phylogenomics^12,19^. Similarly, certain plastome regions identified as unique barcodes are tested and employed in problematic taxa, such as *accD* and *rrn16-rrn23* for yew species^11^ and *psbE-psbL* and *ndhA* intron for *Fagopyrum* species^20^.

Plastome has more variation with a significantly higher resolution of phylogenies than the most often used and predicted genus-specific DNA barcodes, which is beneficial for revealing phylogenetic connections between closely related species^21^. Plastome has been widely applied in plant identification^22^, phylogenetic analyses^16,22–25^ and plant population studies^25^. Even with reference genomes or specific fragments, most taxa, such as Zygophyllum and Tetraena, can build a comprehensive database. The genus *Zygophyllum* L., located in the Arabian Peninsula, has received much interest because of its morphological and anatomical characteristics^26–33^. Most Saudi Arabian *Zygophyllum* taxa were reassigned to *Tetraena*, according to Beier et al.^34^ most recent Tetraena and Zygophyllum taxonomic proposal. In *Zygophyllum* and *Tetraena*, the growth habits, leaf features, floral traits, and fruit shapes are morphologically identical. Beier et al.^34^ employed fruit dehiscence and staminal appendages to distinguish *Zygophyllum* and *Tetraena*. With six genera: *Zygophyllum*, *Fagonia*, *Augea*, *Roepera*, *Tetraena*, and *Melocarpum*, the Zygophylloideae subfamily is currently the largest^34,35^. Sheahan and Chase^36^ examined *rbcL* and *trnL-F* sequences and discovered that *Tetraena* is nested within the large and diverse *Zygophyllum*.

*Tetraena qatarensis* (Hadidi), vernacular ‘Haram’ in Arabic, is an element of the flora of Eastern Saudi Arabia^30^ and a constituent specie of coastal lowland vegetation of Saudi Arabia^37^. El Hadidi^26^ reported the *Z. qatarense* as a new species of Qatar. Hosny^29^ also identified three varieties and thirteen *Zygophyllum* species, including some Saudi Arabian varieties and species. The Arabian Peninsula and Saudi Arabia are home to *Z. qatarense*^26^. According to different authors^27,29,33,38–51^, *Z. mandavillei*, *Z. hamiense* and *Z. qatarense* are three different species, whereas others (Chaudhary^31^) believed that the other two are varieties of *Z. hamiense* because of similar fruit and leaflet morphology. Sheahan and Chase^36^ studied the phylogenetic relationship of Zygophyllaceae based on anatomy, morphology, and *rbcl* gene sequence and found that *Fagonia* is a sister to the Zygophyllaceae family. Interestingly, *Z. fabago* is sister to *Augea* genus while *Z. simplex* might not belong to *Zygophyllum* but at the same time is sister to the *Tetraena* genus. *Zygophyllum simplex* L. is a salt-tolerant plant that belongs to Zygophyllaceae locally (in Saudi Arabia)^38,52^. Later, Sheahan and Chase^36^ selected thirty-six Zygophyllaceae taxa, including fifteen *Zygophyllum* species of regions like Australia, Africa, and Southeast Asia based on plastome genes *rbcL* and *trn-F* (non-coding), investigated the phylogenetic relationship. It was found that *Zygophyllum* is polyphyletic and eventually classified into five clades with high bootstrap values. Furthermore, according to molecular investigations, *Tetraena* is also located within the large, paraphyletic *Zygophyllum*^36^. Later on, these species, with other 33 species, were transferred from *Zygophyllum* to *Tetraena* by Beier et al.^34^ and are currently known as *T. hamiensis* (Schweinf.) Beier & Thulin, 2003; *T. mandavillei* (Hadidi) Beier & Thulin, 2003; and *T. hamiensis* var. *qatarensis* (Hadidi)^34^. According to Ghazanfar and Osborne^53^, *Tetraena* species found in the Arabian Peninsula and Saudi Arabia include *T. qatarensis, T. decumbens*, *T. coccinea*, *T. alba*, *T. propinqua, T. hamiensis*, *T. dumosa*, *T. mandavillei,* and* T. simplex.*

Compared to the phylogenetic tree based on individual barcodes, the full plastomes tree has a greater supporting rate and lesser discrimination^54^. Li et al.^18^ suggested that the complete plastome be used as a super-barcode to identify closely related species accurately. This study sequenced the full plastomes of *Tetraena qatarensis* and *Tetraena* simplex and compared them to previously published complete plastomes of *Zygophyllum* and *Tetraena* species. We analyzed the full plastomes of the *Tetraena* and *Zygophyllum* species, which are difficult to distinguish based on appearance and taxonomy alone. Our study had three objectives: (i) understand whether the plastome can be used as a super-barcode to identify closely related species, (ii) sequence new plastomes from *Tetraena* species and explore polymorphic regions within plastomes, and (iii) assess the discrimination power of plastomes in the genus *Tetraena*. This study's findings may assist in investigating the possibility of using the plastome to differentiate between different *Tetraena* species and serve as a barcode to distinguish between closely related species.

## Results

### General features of *T. hamiensis* var*. qatarensis* and *T. simplex* of plastomes and their comparison to related species plastomes

**Figure 1:**
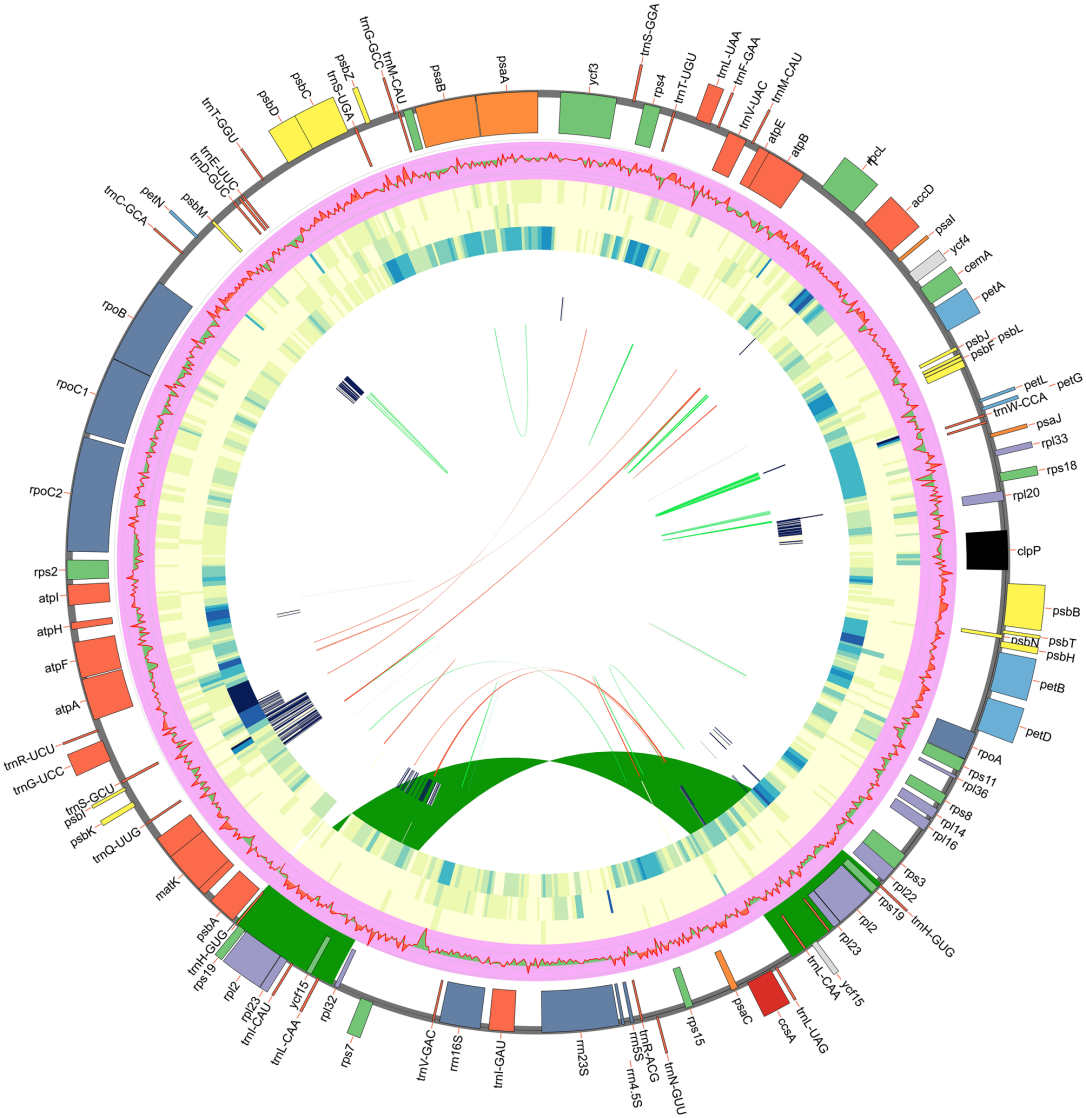
Circos view of complete plastome of *T. hamiensis* var. *qatarensis* with related species*.* Genes shown on the outside outer circle are transcribed counterclockwise and, on the inside, clockwise. (Track 1) the pink color track show guanine-cytosine (GC) skew. The heatmap track from outside to inside is nucleotide diversity (Pi) of *T. hamiensis* var. *qatarensis* with three *Tetraena* species (track 2), five *Zygophyllum* species (track 3), and twelve species plastomes from the family Zygophyllaceae used in this study (track 4). The inner three heatmap tracks show SNP and InDels of *T. hamiensis* var. *qatarensis* with three *Tetraena species* (track 5), five *Zygophyllum* species (track 6), and twelve species plastomes from the family Zygophyllaceae (track 7). Dark green color links show the inverted repeats (IR) region of *T. hamiensis* var. *qatarensis*. The palindromic, forward, and reverse repeats are linked by lines in different colors: forward repeats (green), reverse repeats (light blue), and palindromic repeats (red) color. The heatmap was generated using CIRCOS (http://circos.ca/software/).

The complete sequenced plastomes of *Tetraena* species, *T. hamiensis* var*. qatarensis* (OM809718) and *T. simplex* (OL943588) have typical circular quadripartite structures like other angiosperm plastomes. However, plastome sizes of *T. hamiensis* var*. qatarensis* and *T. simplex* are 106,720 bp and 106,446 bp, respectively, which are smaller than typical angiosperms plastomes; each circular genome is divided into large single-copy (LSC) region (80,964 bp, 80,554 bp), small single-copy (SSC) region (17,416 bp, 17,280 bp) and two inverted repeats (IR) regions (4170 bp, 4306 bp) (Figs. 1, S1 and Table 1). The plastomes were analyzed and compared with eleven other PCD (protein coding region) Zygophyllaceae (three *Tetraena,* five *Zygophyllum,* one *Larrea,* one *Tribulus,* and one *Guaiacum*) species. In all these plastomes, the most significant size was noted for *Tr. terrestris* (158,184 bp) followed by *L. tridentate* (136,194 bp) and *G. angustifolium* (130,809 bp), while the smallest genome size was noted for *Z. fabago*2 (104,590 bp). Similarly, the highest LSC and SSC regions were found in *Tr. terrestris,* 88,878 bp and 17,622 bp respectively and the smallest LSC in *Z. fabago2* (79,170 bp), while the smallest SSC was identified in *Z. xanthoxylon*2 (15,674 bp). Furthermore, the IR region in *T. hamiensis* var*. qatarensis* and *T. simplex* are 4170 bp and 4306 bp respectively; among the other species of *Tetraene* and *Zygophyllum,* the IR regions were found in a similar range between 4288 bp (*Z. xanthoxylon*1) to 4669 bp (*Z. fabago*1). Interestingly, the smallest IR region was found in *T. hamiensis* var*. qatarensis.* The GC content in both sequenced plastomes of *T. hamiensis* var*. qatarensis* and *T. simplex* were 33.6% and 33.9% respectively. The highest GC content (35.8%) was determined in *Tr. terrestris* plastome.

**Table 1:** Summary of all 13 plastomes.

	*T. hamiensis var. qatarensis*	*T. simplex*	*T. mongolica1*	*T. mongolica2*	*T. mongolica3*	*Z. fabago1*	*Z. fabago2*	*Z. fabago3*	*Z. xanthoxylon1*	*Z. xanthoxylon2*	*L. tridentata*	*Tr. terrestris*	*G. angustifolium*
Size (bp)	106,720	106,446	106,288	106,259	106,081	108,695	104,590	104,984	105,423	109,577	136,194	158,184	130,809
Overall GC contents	33.6	33.9	33.6	33.6	33.6	33.7	33.9	33.9	34.1	33.8	35.1	35.8	35.6
LSC size in bp	80,964	80,554	80,458	80,390	80,291	82,459	79,170	79,696	79,877	83,347	81,536	88,878	81,554
SSC size in bp	17,416	17,280	17,200	17,255	17,062	17,064	16,778	16,413	16,645	15,674	15,958	17,622	13,767
IR size in bp	4170	4306	4315	4307	4315	4669	4321	4413	4288	4514	19,350	25,842	17,744
Protein coding regions size in bp	47,091	46,598	46,746	47,406	47,514	46,977	46,359	47,268	47,229	47,172	58,458	78,774	58,485
tRNA size in bp	2461	2561	2491	2424	2505	2508	2504	2494	2505	2571	2660	2807	2782
rRNA size in bp	4524	4523	4523	4523	4520	4514	4536	4514	4288	4514	9038	9046	9037
Number of genes	106	105	104	105	106	104	106	105	106	107	125	130	125
Number of protein coding genes	69	68	67	69	69	67	69	68	68	69	81	84	80
Number of rRNA	4	4	4	4	4	4	4	4	4	4	8	8	8
Number of tRNAs	34	33	33	32	33	33	33	33	33	34	35	37	37
Genes with introns	9 + 5	10 + 6	10 + 6	7 + 5		8 + 5	7 + 6		10 + 6	8 + 6	13 + 7	13 + 8	13 + 8

### Gene content and gene loss in *Tetraena* plastomes

The gene content of the Zygophyllaceae plastomes varied considerably. These plastomes contained 67–84 protein-coding genes, 4–8 rRNA genes, and 32–37 tRNA genes (Fig. 1; Table 1). The number of genes annotated in a plastome ranged from 104 (*T. mongolica*1) to 130 (*Tr. terrestris*). The plastomes *T. hamiensis* var. *qatarensis* and *T. simplex* sequenced in this study had 106 and 105 genes including four rRNA genes, 34 and 33 tRNA genes, and 69 and 68 protein-coding genes, respectively (Table S1). In both plastomes, the protein-coding region, tRNA, and rRNA lengths were 46,598–47,091 bp, 2461–2561 bp, and 4523–4524 bp, respectively (Table 1). In most higher plants the rRNA genes are located in the IRs region however, these are present in the SSC region *Tetraena* and *Zygophyllum* species plastomes. Subsequently, the copy number of rRNA genes changes from 2 to 1. We compared the five *Tetraena*, five *Zygophyllum*, *Tribulus*, *Larrea*, and *Guaiacum* plastomes. We found that all *ndh* genes encoding subunits of NADH oxidoreductase were lost in all *Tetraena* and *Zygophyllum* species except *Z. fabago*1 and *Z. xanthozylon*2 where one copy of *ndhI* retain (Fig. S2). Similarly, one copy of *ndhE* gene was also detected in the *Z. xanthozylon*2 plastome. These genes are usually located in SSC and IR regions. Moreover, *rpl12*, *ycf2*, *ycf1*, and *infA*, common chloroplast genes, were lost in all *Tetraena* and *Zygophyllum* plastomes. Furthermore, the *ycf15* gene was absent in *T. simplex*, *T. mongolica1*, *L. tridentata*, *Tr. terrestris* and *G. angustifolium* plastome. Notably, the *accD* was absent in *L. tridentata* and *G. angustifolium* plastomes. Two copies of *rps19*, *rpl2* and *rpl23* genes were found in all *Tetraena* and *Zygophyllum* species while one copy of *ndhB*, *rrn4.5*, *rrn16*, *rrn5* and *rrn24* were lost with the contraction of inverted repeat regions (Fig. S2).

In both *T. hamiensis* var. *qatarensis* and *T. simplex* about 13.40% and 14.7% of the functional genes contain introns, including 5 and 7 tRNA, 9 and 10 protein-coding genes, respectively and only one gene *ycf3* contains two introns (Table S2). Interestingly, in both *T. hamiensis* var. *qatarensis* and *T. simplex* and other *Zygophyllum* and *Tetraena* plastomes, we observed the absence of *clpP* and *rps12* three end introns. Observation of loss of both introns represents the first documented loss within the Zygophyllaceae plastomes. Our study revealed that the loss of the *clpP* and *rps12* introns are limited to the loss of large IR regions in these plastomes with only one exception where *rps13* both introns were detected in *Z. fabago*3 (MK341052) plastome. Furthermore, the length of these introns ranges from 458 bp (*trnL-UAA*) to 2579 bp (*trnK-UUU*). The *trnA-UGC* gene was found with one intron in *T. simplex;* however, in *T. hamiensis* var. *qatarensis,* it was found to be an intron-less gene (Table S2).

### Functional repeats analysis

**Figure 2:**
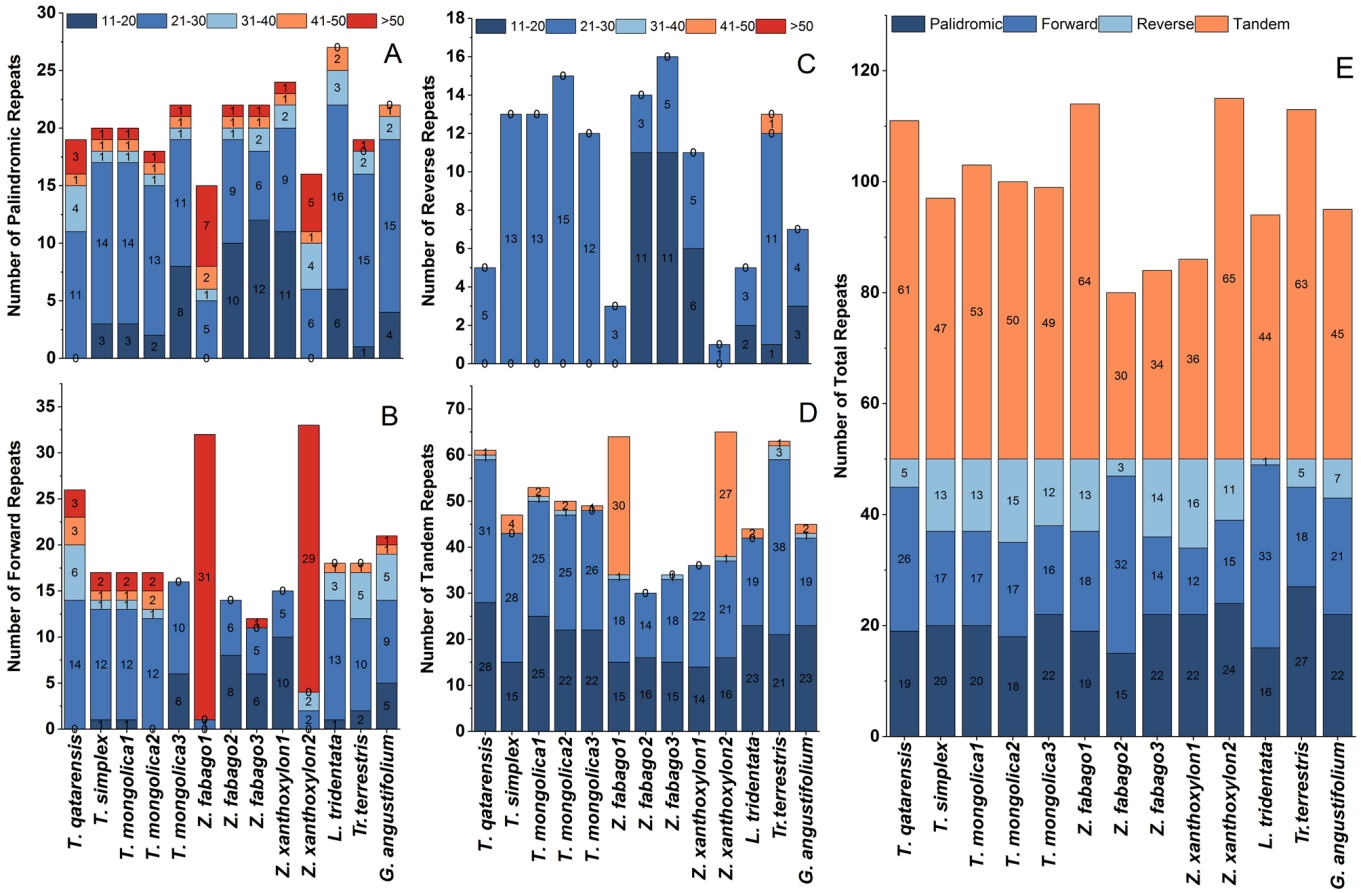
Repetitive sequences in *T. hamiensis* var*. qatarensis*, *T. simplex,* and related species plastomes. (**A**) Lengthwise frequency of palindromic repeats in plastomes, (**B**) Lengthwise frequency of forward repeats, (**C**) Lengthwise frequency of reverse repeats, (**D**) Lengthwise frequency of tandem repeats, and (**E**) Total number of repetitive sequences.

**Figure 3:**
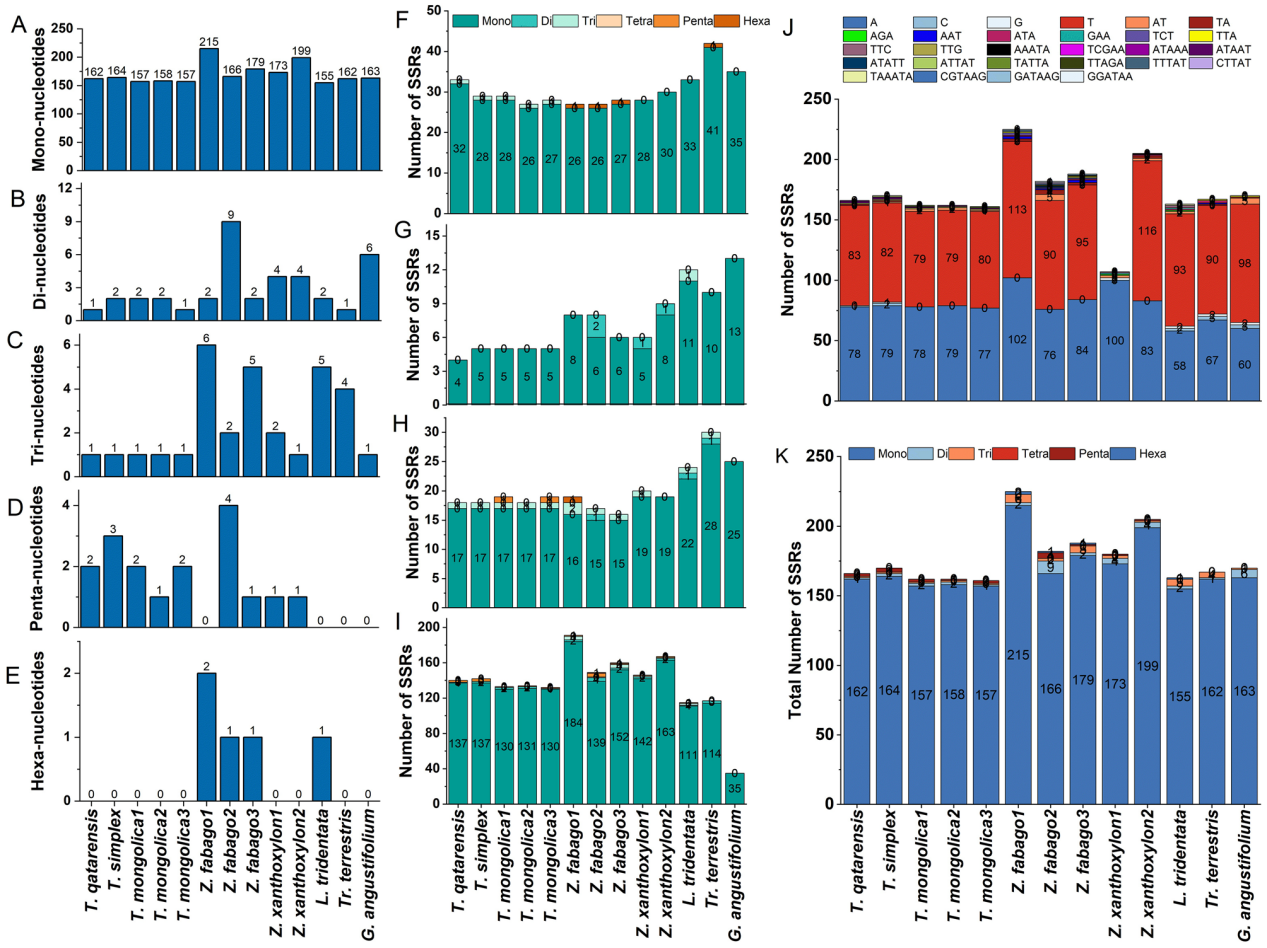
Simple sequence repeats (SSRs) in *T. hamiensis* var*. qatarensis*, *T. simplex,* and *related* species plastomes. (**A**) Mono-nucleotides SSRs (**B**) Di-nucleotides SSRs, (**C**) tri-nucleotides SSRs, (**D**) Penta-nucleotides SSRs, (**E**) hexa-nucleotides SSRs, (**F**) number of SSRs in protein-coding region, (**G**) number of SSRs in inverted repeats region, (**H**) number of SSRs in inverted repeats region small single-copy region, (**I**) number of SSRs in the large single-copy region, (**J**) SSR motif frequency in plastomes, (**K**) total number of SSRs in plastomes.

In functional repeat analysis, various repetitive sequences in plastomes of *T. hamiensis* var*. qatarensis* and *T. simplex* and other related species were determined (Figs. 1, 3). Total of 19 palindromic, 26 forward, five reverse and 61 tandem repeat sequences were identified in *T. hamiensis* var*. qatarensis* plastome. The *T. simplex* plastome contained 20 palindromic, 17 forward, 13 reverse and 47 tandem repeat sequences (Fig. 2E). The *L. tridentata* plastome contained the highest number of palindromic repeats (27), while the *Z. fabago*2 plastome had the lowest (15) palindromic repeat sequences. The Z. *xanthoxylon* plastomes had maximum forward repeat sequences (33) and minimum reverse repeats (1). The highest number of reverse repeats was identified in plastome of *T. mongolica2* (15). The minimum number of forwarding repeats (12) was found in *Z. fabago3.* Similarly, in *Z. xanthoxylon2* plastome, 65 tandem repeats were determined, which is the highest in all the plastomes included in the present study (Fig. 2D).

Furthermore, maximum number of palindromic, forward and reverse repeat sequences in all plastomes identified were 21–30 bp in length except in plastomes of *Z. fabago*1, *Z. fabago*2 and *Z. xanthoxylon*1 in which the highest number of mentioned repetitive sequences were 11–20 bp in length (Fig. 2). Among all plastomes the forward repeat sequences of > 50 bp length were most in *Z. Fabago1* (31) and *Z. xanthoxylon2* (29) (Fig. 2A). The highest frequency of tandem repeat sequences was identified in 1–30 bp length, with highest number (28) in *T. hamiensis* var*. qatarensis* (Fig. 2D). As noted for *Z. Fabago1* (31) and *Z. xanthoxylon2* (29), in forward repeat sequences, the highest number of tandem repeats were also maximum in > 45 bp length.

### Simple sequence repeats (SSRs) analysis

The SSRs were analyzed in all plastomes included in the study (Fig. 3). Like other functional repeat sequences, SSR numbers differed in all these plastomes. Total number of SSRs ranged from 162 (*T. mongolica*3) to 225 (*Z. fabago*1). The total SSRs in sequenced plastomes of *T. hamiensis* var*. qatarensis* and *T. simplex* were 166 and 170 respectively (Fig. 3K). Moreover, *T. hamiensis* var*. qatarensis* have 162 mono, one di, one tri and two penta-nucleotides SSRs while *T. simplex* has 164 mono, two di, one tri and three penta-nucleotides SSRs. Surprisingly, no tetra-nucleotides SSRs were identified in all plastomes included in this study. Unlike the other plastomes, the SSR numbers were not correlated with the size of plastomes. The highest sized plastome (*Tr. terrestris*) has a similar number of SSRs as the smaller-sized plastome (*T. hamiensis* var*. qatarensis*). Interestingly, the highest number of total SSRs were determined in the plastome of *Z. fabago*2 which has the smallest size compared to plastomes.

As for the complete plastomes the SSRs numbers were also determined in various parts of the plastomes like LSC, SSC, IR and protein coding regions. LSC regions of all plastomes had the highest number of SSRs (Fig. 3I). The plastomes of *T. hamiensis* var*. qatarensis* and *T. simplex* have 140 (84% of total SSRs) and 142 (83% of total SSRs) in LSC regions respectively, in which 137, 137 mono, 1,2 di and 2, 3 pentanucleotides were identified respectively. The sequenced plastomes contained 18 (17 mono and one tri nucleotides SSRs each) in the SSC region (Fig. 3H). Furthermore, the IR regions of the sequenced plastomes of *T. hamiensis* var*. qatarensis* and *T. simplex* have 4 and 5 (only mono-nucleotides) SSRs. interestingly the plastomes of *L. tridentate* and *Tr. terrestris* have 11 and 10 SSRs in IR regions (Fig. 3G). The difference among the SSR numbers in IR regions is attributed to the IR sizes in these plastomes. The highest number of SSRs in protein-coding region was detected in *Tr. terrestris* (41), *L. tridentate* (33) followed by *T. hamiensis* var*. qatarensis* (33). At the same time, lowest was determined in *T. mongolica*2 *T. mongolica*3 *Z. fabago*1 and *Z. fabago*2 (26 each) (Fig. 3F).

### Comparative analysis and divergence

Comparison of complete plastomes of *T. hamiensis* var*. qatarensis*, *T. simplex* and 11 other closely related species were performed. The 13 complete plastomes and 58 shared protein coding genes were aligned and compared to determine the average pairwise distance among these species using *T. hamiensis* var*. qatarensis* as a reference (Table S3). *Larrea tridentata* (0.177384) showed the highest average pairwise distance, followed by *Tribulus terrestris* (0.163060). The minimum average pairwise distance was exhibited by *T. mongolica*1 (0.023) and *T. simplex* (0.027). Among the 58 shared protein coding genes, the *atpF, clpP psbH, rpl20, rpl22*, and *rps2* were most divergent (Table S4). The highest average pairwise sequence divergence was determined for *clpP* gene across all compared species. Still, the highest divergence was recorded in *Tribulus terrestris* (0.642) and (0.505) followed by *Z. fabago*3 (0.177) and *Z. xanthoxylon*2 (0.172) (Table S4).

Furthermore, the values of nucleotide diversity (Pi) were determined in plastomes *T. hamiensis* var*. qatarensis*, *T. simplex* and other related species (Figs. 1, S3). The genomes were aligned in three different groups: (i) Two currently sequenced and three *Tetraena* plastomes, (ii) Two sequenced and five *Zygophyllum* plastomes, (iii) all plastomes included in the study to better evaluate and understand the nucleotide diversity (Pi). The nucleotide diversity (Pi) values within 200 bp window size and 100 bp step size across these plastomes vary from 0 to 0.2 (Fig. S3A), 0 to 0.3 (Fig. S3B) and 0 to 0.4 (Fig. S3C). Only 2 variable loci (*psbE-PetL, clpP*) were found with Pi > 0.1 in *Tetraena* plastomes while in *Tetraena*-*Zygophyllum* 4 variable loci (*atpF, clpP, rps15-trnL, ycf2*) were found with Pi > 0.2. In overall comparison of all 13 plastomes, several variable loci were identified having Pi value > 0.2, in which the highest Pi value of 0.323 was noted for (*rps12*) followed by (*rpoC1*) having Pi 0.285 (Fig. S3C).

**Figure 4:**
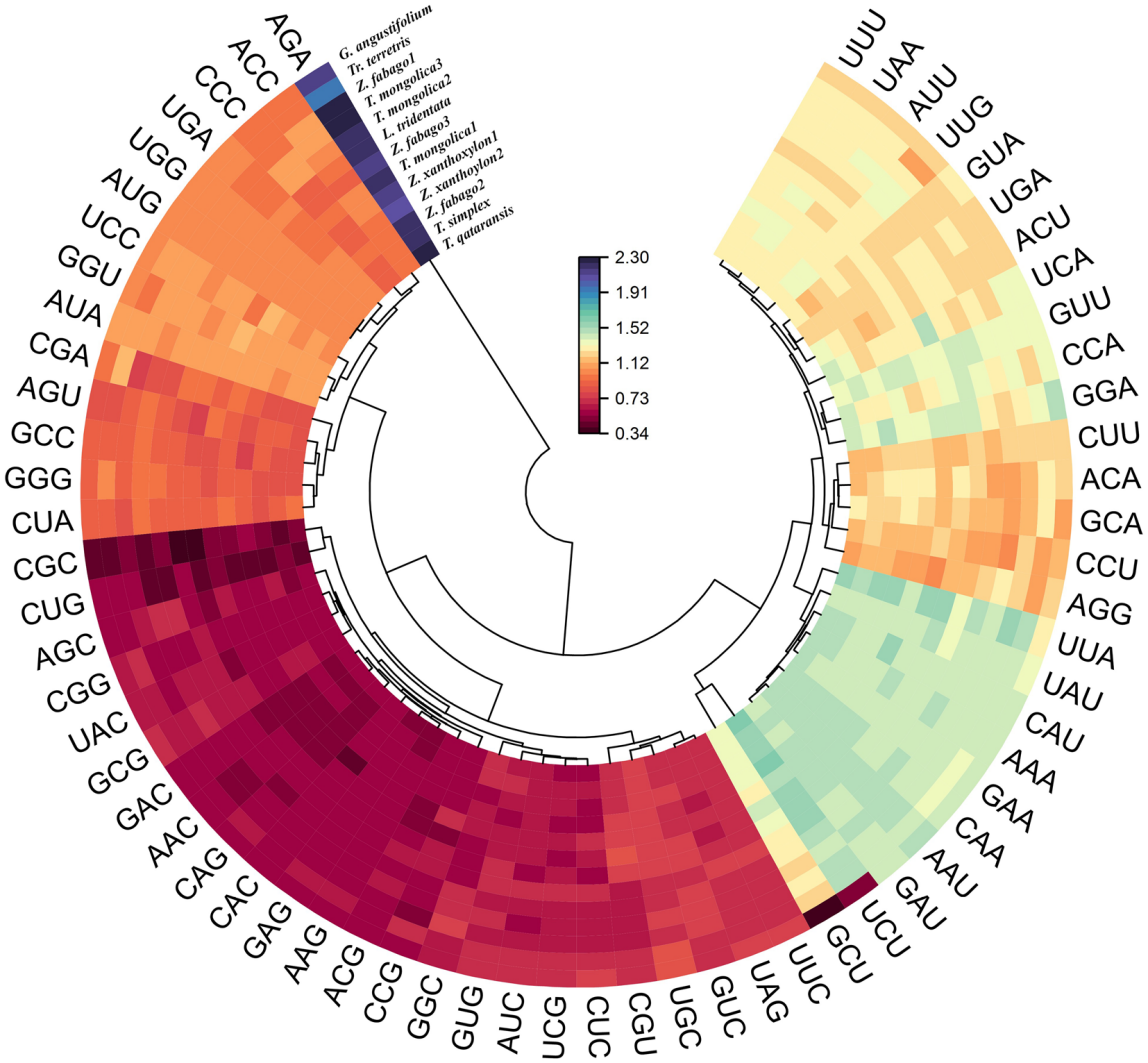
Heatmap plot of codon distribution of all shared protein-coding genes in *T. hamiensis* var*. qatarensis*, *T. simplex,* and *related* plastomes. Color key: red indicates lower, yellow means moderate, while black indicates higher RSCU values.

Similarly, the codon usage frequency *T. hamiensis* var*. qatarensis* and *T. simplex* protein-coding genes were determined. The protein-coding genes in *T. hamiensis* var*. qatarensis*, and *T. simplex* were composed of 20,122 and 27,231 codons respectively (Table S5). The highest codon used in *T. hamiensis* var*. qatarensis* plastome was ATT (884) which codes for isoleucine. In *T. simplex* plastome, the maximum used codons were AAA (1142) which codes for lysine and AAG (1142) which codes for isoleucine (Table S5). Furthermore, Relative Synonymous Codon Usage (RSCU) was also analyzed for all 13 plastomes (Fig. 4). Surprisingly, almost all of these codons had half-synonymous codons that ended in A or T and had high RSCU values. The other half had low RSCU ended with C and G. The third codon position showed biased codon usage toward A and U, which is consistent with the pattern seen in the majority of angiosperm plastomes^55^.

Genome divergence in all 13 plastomes was characterized by mVISTA using *T. hamiensis* var*. qatarensis* as a reference genome (Fig. S4). The divergence in LSC region was higher than SSC and IRs. In all LSC, SSC, and IRs, the non-coding region diverges more than the coding region. A significant divergence was identified among the protein-coding regions in *matK, atpA, atpF, rpoC1, rpoC2, rpoB, rps11, petB, petD* and *accD* genes in LSC region. The divergence in these genes was lower in *Tetraena* plastomes compared to *Zygophyllum* and other related plastomes. The LSC region non-coding regions *trnQ-trnR*, *trnC-trnT*, *trnM*, *trnT-trnV* had higher divergence in all plastomes than *T. hamiensis* var*. qatarensis* (Fig. S4). Most of the SSC region's divergence was found in non-coding regions like *trnV-trnI* and *trnL-trnI*. Interestingly, in IR regions the divergences in *rps19* and *rpl2* genes were only detected in *Tr. terrestris* and* L. tridentata.*

The SNPs (single nucleotide polymorphism) and InDels were evaluated in the complete plastome of *T. hamiensis* var*. qatarensis* to that of all other plastomes (Fig. 1, Table 2). 5506 SNPs and 1600 InDels were detected in plastome *T. hamiensis* var*. qatarensis* to *T. simplex.* The minimum number of SNPs and InDels were detected in *T. hamiensis* var*. qatarensis* to *T. mongolica*3 (4812 SNPs) and *T. hamiensis* var*. qatarensis* to *T. mongolica*2 (1781 InDels) respectively. In *Zygophyllum* species the highest SNPs and InDel were identified in plastome *T. hamiensis* var*. qatarensis* to *Z. fabago*1 13,144 and 13,739 respectively. Overall, the highest number of SNPs were identified in plastome *T. hamiensis* var*. qatarensis* to *L. tridentata* (28,348) The highest InDels were 49,513 in *T. hamiensis* var*. qatarensis* to *Tr. terrestris*. Similarly, the SNPs and InDels were also evaluated in different parts of plastomes. In plastome *T. hamiensis* var*. qatarensis* to *T. simplex* the number of SNPs and InDels were 4,274 and 1455 (LSC), 792 and 168 (SSC), 132 and 8 (IR) and 3076 and 911 (protein coding region) respectively (Table 2). IRs had a minimum number of SNPs and InDels in all plastomes in all parts of the plastomes.

**Table 2:** SNPs and indels evaluation in the *T. hamiensis* var*. qatarensis* plastome to other related plastomes.

S. no.	Genomes	Complete plastome		LSC		SSC		IRs		PCD	
		SNP	Indel	SNP	Indel	SNP	Indel	SNP	Indel	SNP	Indel
1	*T. qatarensis*–*T. simplex*	5506	1600	4274	1455	792	168	132	8	3076	911
2	*T. qatarensis–T. mongolica1*	4918	1926	4552	1787	56	184	4	0	1856	797
3	*T. qatarensis–T. mongolica2*	4874	1781	4528	1723	40	95	2	0	1852	911
4	*T. qatarensis–T. mongolica3*	4812	2091	4446	1904	14	161	2	0	1824	1313
5	*T. qatarensis–Z. fabafo1*	13,144	13,739	9766	9288	2212	3601	378	504	3665	1116
6	*T. qatarensis–Z. fabafo2*	12,932	10,936	9619	7465	2134	2810	398	190	3506	1540
7	*T. qatarensis–Z. fabafo3*	12,932	10,936	9592	7361	2214	2979	388	267	3626	1325
8	*T. qatarensis–Z. xanthoxylon1*	12,638	13,275	9270	6726	2084	2773	418	348	3486	1228
9	*T. qatarensis–Z. xanthoxylon2*	12,470	10,307	9278	9658	1716	2350	454	1368	3632	2047
10	*T. qatarensis–Tr. terrestris*	25,902	67,155	20,906	17,481	8294	10,384	508	23,191	8204	33,163
11	*T. qatarensis–L. tridentata*	28,348	49,513	22,294	14,941	11,702	5280	874	15,727	8424	17,415
12	*T. qatarensis–G. angustifolium*	27,702	43,460	21,610	15,613	10,432	5670	926	13,904	8474	17,770

### Unusual contraction of IR region detected in *Tetraena* plastomes

**Figure 5:**
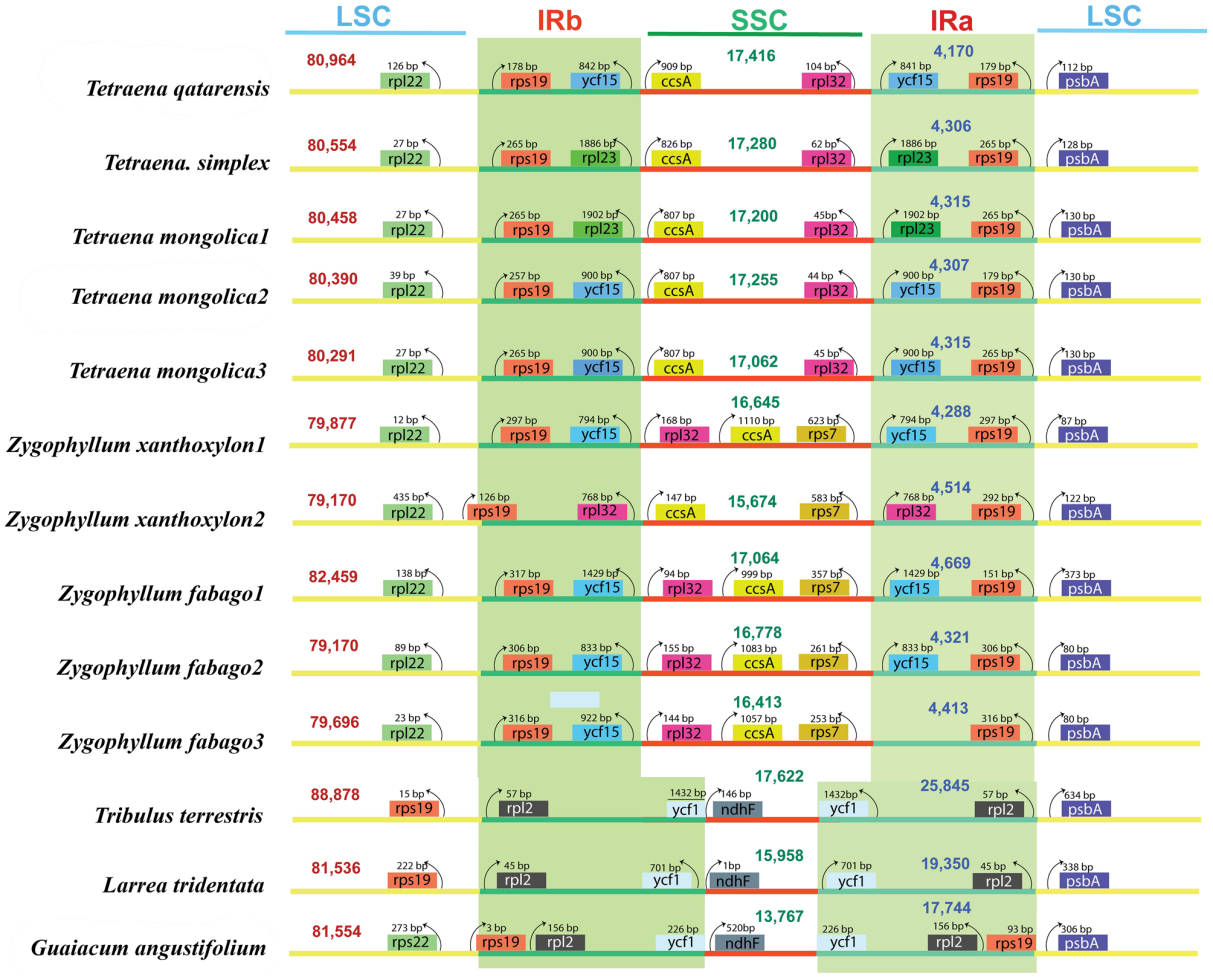
Distances between adjacent genes and junctions of the small single-copy (SSC), large single-copy (LSC), and two inverted repeats (IR) regions among *T. hamiensis* var*. qatarensis*, *T. simplex,* and *related* plastomes. Boxes above and below the primary line indicate the adjacent border genes. The figure is not scaled regarding sequence length and only shows relative changes at or near the IR/SC borders.

The IR regions in a plastome are considered the most preserved portions.. The IR regions contraction and expansion determine the variability in plastome sizes and are common evolutionary events. Herein, four IR junctions J^LB^ (LSC/IRb), J^SB^ (SSC/IRb), J^SA^ (SSC/IRa), and J^LA^ (LSC/IRa), boundary comparison analysis was performed, to determine the contraction and expansion events (Figs. 5, S5). The sub-familial members (*Tetraena* and *Zygophyllum* belonging to Zygophylloideae) showed high contraction in IR regions compared to the other family members *L. tridentata, G. angustifolium* and other family members *Tr. terrestris,* resulting in gene loss, gene order changes and rearrangements in the plastomes. The IR lengths in *L. tridentata, G. angustifolium* and *Tr. terrestris,* are 19,350, 17,744 bp and 25,842 bp respectively while the IR lengths in the sequenced plastomes *T. hamiensis* var*. qatarensis*, *T. simplex* is 4170 bp and 4306 bp, respectively. Furthermore, the location of genes at these junctions was also determined. The contraction of IR regions in *Tetraena* and *Zygophyllum* species including the two sequenced plastomes, resulted in altered gene locations. At J^LB^ junction, all plastomes have noted variation in *rpl22* (LSC) and *rps19* (IRb) locations. The *rpl22* gene is located 126 bp and 27 bp away from JLB in LSC region in plastomes *T. hamiensis* var*.* qatarensis and *T. simplex* respectively. Similarly, the *rpl19* gene is 178 bp and 265 bp away from JLB in the IRb region in plastomes *T. hamiensis* var*. qatarensis* and *T. simplex*, respectively (Fig. 5). Moreover, in *T. hamiensis* var*. qatarensis* at the J^SB^ *ycf15* (842 bp away in IRb) and *ccsA* (909 bp away in SSC) are located while in *T. simplex* and *T. mongolica*1, the *ycf15* gene is lost so *rpl23* takes this position into IRb (1886 bp away) at J^SB^. Similarly, in *Z. xanthoxylone*2, the position of *the rps19* gene is different than in other plastomes (Figs. 5, S5). In *Tr. terrestris* and *L. tridentata* plastomes, the location of genes has changed at J^LB^; the rps19 gene shifted from IRb to LSC in both plastomes with slight variation in position while in *G. angustifolium* it was like *Tetraena* and *Zygophyllum* plastomes (Fig. 5). Similarly, variations have been observed at JSB among *Tetraena*, *Zygophyllum* and other species. In *Z. xanthoxylon*1 the *rpl32* gene is present in IRb unlike other plastomes at J^SB^. Furthermore, due to full lengths of IRs in *Tr. terrestris* and *L. tridentata* and *G. angustifolium* the genes arrangements at J^SB^ and J^SA^ are different from *Tetraena* and *Zygophyllum* plastomes. In these three plastomes the *ycf1* genes are in IRb and the *ndhF* gene is located in SSC at J^LB^ (Fig. 5).

### Phylogenetic analysis and divergence time

We conducted phylogenetic analyses using ML, NJ, MP, and BI based on five distinct data sets, such as complete plastomes, to examine evolutionary relationships between *Tetraena* and *Zygophyllum* species and preliminarily assess species boundaries (Fig. S6), 58 concatenated protein-coding genes (Fig. S7), *matK* gene (Fig. S8)*, rbcL* gene (Fig. S9) and *cssA* gene (Fig. S10) independently. Protein-coding genes, *matK*, *rbcL* and *cssA* genes showed almost similar topologies in all the trees resulted from complete plastomes.

**Figure 6:**
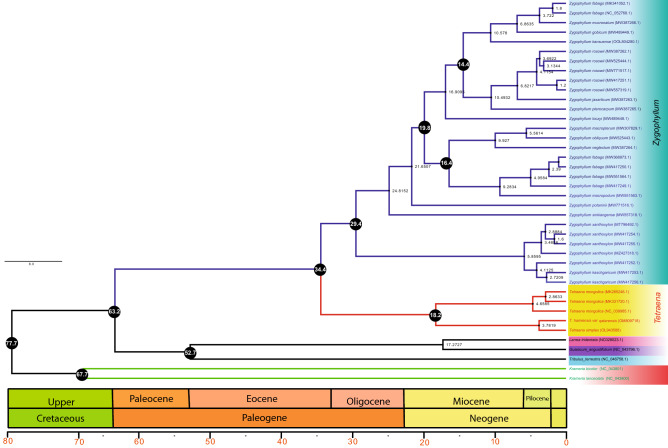
The maximum credible molecular chronogram (time tree) of *Tetraena* from BEAST is based on whole plastomes, with branch lengths proportional to time and lognormal fossil-based calibrations. The GTR + G substitution model was used with four rate categories and a Yule tree speciation model was applied with a lognormal relaxed clock model in BEAST. Different color branches represent subgroupings in Zygophyllaceae. The 95% highest posterior density credibility intervals for the node ages in black circles (mya). Numbers indicate date estimates for different nodes. A geological time scale is shown at the bottom of the figure.

**Figure 7:**
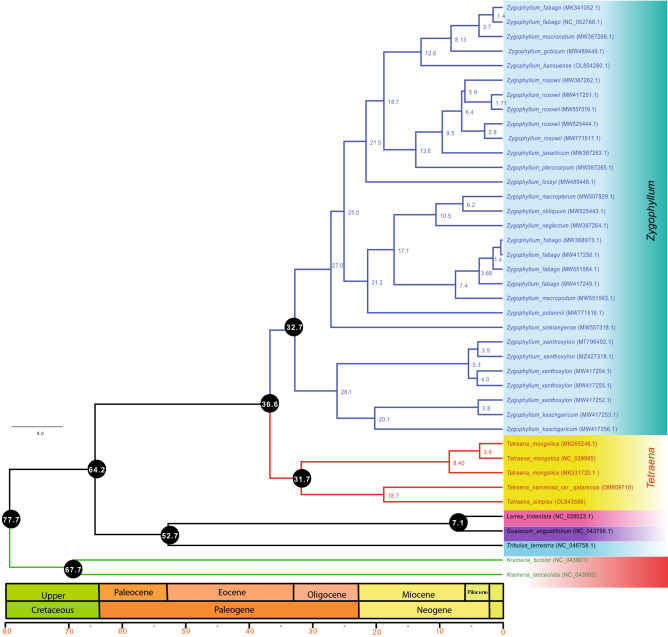
Divergence time estimates of Zygophyllaceae based on 58 protein-coding shared genes from *T. hamiensis* var. *qatarensis*, *T. simplex* and related species. The GTR + G substitution model was used with four rate categories and a Yule tree speciation model was applied with a lognormal relaxed clock model in BEAST. Different color branches represent subgroupings in Zygophyllaceae. The 95% highest posterior density credibility intervals for the node ages in black circles (mya). Numbers indicate date estimates for different nodes. A geological time scale is shown at the bottom of the figure.

The reconstructed phylogenies from the complete plastome (Figs. 6, S6) and shared protein-coding genes (Figs. 7, S7) datasets showed that *Zygophyllum* and *Tetraena* clustered into two main clades and *Zygophyllum* species were divided into two further clustered. The first *Zygophyllum* cluster comprised *Z. kaschgaricum* and *Z. xanthoxylon* with high bootstrap values. The second cluster was divided further into various clades. According to both trees (complete plastomes and shared protein-coding genes) *Z. rosowii* cluster with *Z. jaxarticum* and *Z. pterocarpum*. However, *Z. fabago* showed different results and *Z. fabago* (MK341052.1) and *Z. fabago* (NC_052768.1) clustered with *Z. mucronatum*, *Z. gobicum* and *Z. kansuense* in same clades (i). In contrast, the other Fabago species *Z. fabago* (MW417250.1), *Z. fabago* (MW551564.1) and *Z. fabago* (MW417249.1) are clustered with *Z. macropdum* in another clade (III) based on concatenated shared genes and complete plastomes. Similar results were obtained from trees based on *matK*, *rbcL* and *cssA* genes data sets. The *Tetraena* species were clustered into one clade sister to the other *Zygophyllum* species. Within the *Tetraena* species, the *T. mongolica* formed a monophyletic group and *T. hamiensis* var. *qatarensis* and *T. simplex* were clustered into one group based on complete plastomes, proteins coding genes and *matK* datasets. However, based on *rbcL* and *ccsA* genes *T. simplex* and *T. hamiensis* var. *qatarensis* were not clustered into one clade. In *cssA* based tree *T. hamiensis* var. *qatarensis* was clustered with *T. mongolica* (NC039985) while *T. simplex* was found in a separate clade. The *L. tridentata* species clustered with *G. angustifolium* species, and *Tr. terrestris* was found sister clade. The two outgroups from *Krameria* species had a far phylogenetic relationship to *Zygophyllum*. Overall, the phylogenetic trees based on the above data sets show almost the same result except for the *rbcL* and *cssA* genes, which show variation in some species.

The backbone topology in *Zygophyllaceae* was largely congruent among ML, NJ, MP, and the BEAST2 analyses, receiving moderate to high bootstrap support and posterior probabilities. The final alignment for complete plastome and 58 shared concatenated genes had 77,526 bp and 42,229 bp, respectively. Phylogenetic trees based on complete plastomes (Fig. 6) and protein-coding genes (Fig. 7) showed similar trees and divergence time estimations with few differences. Based on complete plastomes, we estimated the stem node age of Zygophyllaceae and Krameriaceae at 77.7 Ma (95% HPD: 77.2–78.0), i.e., Cretaceous. The stem age of Zygophylloideae was found at 64.2 Ma (95% HPD: 57.2–72.6), while the crown age was estimated at 36.6 Ma (95% HPD: 32.9–43.8). Furthermore, *Tetraena* species diverged from *Zygophyllum* species in Eocene at 36.6 Ma (95% HPD: 32.9–43.8). On the other hand, tree divergence time based on concatenated protein-coding genes showed similar results in the case of stem node age of Zygophyllaceae and Krameriaceae was estimated at 77.7 Ma (95% HPD: 77.1–78.6), while the crown age of Zygophylloideae was very different from whole plastome tree and estimated 63.2 Ma (95% HPD: 53.8–70.6). Similarly, the *Tetraena* species were estimated at 34.4 Ma (95% HPD: 28.2–47.7) from the *Zygophyllum* species. Furthermore, in both trees based on complete plastomes and protein-coding genes, *T. hamiensis* var. *qatarensis* and *T. simplex* diverged from other *T. mongolica* at 31.7 and 18.2 Ma.

## Discussion

*T. hamiensis* var. *qatarensis* and *T. simplex* plastomes are almost similar in size to previously reported *Tetraena* and *Zygophyllum* plastomes^56^ and significantly shorter than other angiosperms^57–59^. Both *Tetraena* plastomes had a quadripartite structure, standard in vascular plants^60^, and the GC content was similar to that of many other angiosperm species^59,61^. Plastomes of most angiosperms are 120–160 kb long, but the plastomes of *T. hamiensis* var. *qatarensis* and *T. simplex* are between 106,720 and 106,446 bp long. The LSC sections of most angiosperms are around 80–90 kb long, whereas the SSC regions are about 16–27 kb long, and the size of two IRs is approximately 20–28 kb long. The sizes of *T. hamiensis* var. *qatarensis* and *T. simplex* LSC and SSC do not differ significantly from those of most angiosperms, with the most noticeable difference being in two decreased IRs by approximately 16–24 kb in size. As previously reported in *Tetraena* and *Zygophyllum* species plastomes, the shrinking of IRs is primarily responsible for the lower sizes of plastomes in these two *Tetraena* species^56^. Although even smaller plastomes have been reported in non-photosynthetic, parasitic plants, such as *Rhizanthella gardneri* (*Orchidaceae*) with just 59,190 bp, *Zygophyllum* and *Tetraena mongolica* contain genomes on the small end of the range^56,62^.

Several plastomes are substantially smaller than most other plants, even though the chloroplast genome is a very conservative^63^. The most common reports of small plastomes come from investigations of parasitic plants, such as *Taxillus chinensis* and *Taxillus sutchuenensis* in the Loranthaceae family of Santalales^64^ *Epifagus virginiana* in Orobanchaceae family^65^, *Cuscuta chinensis* and *C. japonica* in Convolvulaceae family^66^. Some gymnosperms, such as *Welwitschia mirabilis* in the Welwitschiaceae of the Welwitschiales and *Gnetum ula* in the Gnetaceae of the Gnetales^67,68^, have smaller plastomes. Except for *Astragalus membranaceus*, which has a plastome of roughly 124 kb owing to the lack of an IR, non-parasitic angiosperms rarely have a plastome less than 130 kb^69^. The reduction in the size of SSCs was linked to the contraction of plastomes in other plant species^70,71^. LSC and SSC sizes drop slightly in both *Tetraena* plastomes, whereas IR lengths decrease considerably, as found before in other Zygophyllaceae taxa. As a result, these two new plastomes and others from the Zygophyllaceae family might be used as new models to study plastome structure and size evolution.

Aside from genome size, the number of genes in *T. hamiensis* var. *qatarensis* and *T. simplex*, as well as other related *Tetraena* and *Zygophyllum* plastomes, ranges from 104 to 106, which is lower than other Zygophyllaceae members such as *L. tridentata* and *Tr. terrestris* (125–130 respectively) (Table 1) and other land plants^72,73^. When five *Tetraena* and five *Zygophyllum* plastomes are compared to three other Zygophyllaceae species, four rRNA genes were found in the SSC region in these *Tetraena* and *Zygophyllum* plastomes, resulting in the reduction of copy number of rRNA genes (Table 1). Moreover, the contraction and expansion in IR regions forced the transferring of some genes to the LSC region and as result the IR regions became the single copies. Most of the SSC region's genes assigned to the IR regions duplicated, changing the number of genes, growing the LSC region's size, and shrinking the SSC region^70^. Furthermore, as previously observed, some genes within the NADH dehydrogenase complex (i.e., *ndh* genes) generally found in the SSC and IRs region encoding NADH oxidoreductase subunits are missing plastomes^74^. Furthermore, both *T. hamiensis* var. *qatarensis* and *T. simplex* plastomes lack *rps16*, *rpl12*, *ycf2*, and *infA*, found in most angiosperm plastomes. All of the factors above might contribute to the IRs area's shrinkage. This is uncommon in non-parasitic plants^75^. However, it has been found in *Najas*^76^, several orchid species^77^, Pinaceae^67^, and gametophytes^67,78^. The NADH dehydrogenase complex has role in photosynthesis against the environmental stress in plant plastids. Although infrequent, *ndh* gene deletion or pseudogenization is expected in the plastomes of photoautotrophic seed plants of many lineages^79^. The phenomena described in which plant plastid *ndh* genes were specifically deleted, and nuclear-encoded NDH subunits were expressed in Pinaceae^67^, Orchidaceae^80^ gametophytes^78^ and Geraniales^16,81^. Plants that thrive in drought (arid and semi-arid) conditions must adapt to their surroundings more than others. The current finding reveals the loss of 11 *ndh* genes in the plastomes of *T. hamiensis* var. *qatarensis* and *T. simplex*; it is unclear whether plastid-encoded *ndh* genes have been lost entirely or functionally moved to the cell nucleus for these two Zygophylloideae species. Similar results were seen in the plastid genome of the highly drought-resistant saguaro cactus (*Carnegiea gigantea*), where all *ndh* genes were absent or non-functional^82^.

The current study assessed the SNP and InDel numbers in all plastomes compared to *T. hamiensis* var. *qatarensis* in whole genomes and different regions, i.e., LSC, SSC IRs, and protein coding region (Table 2). Minimum SNPs were identified in *T. qatarensis–T. mongolica*3 (4812), while minimum InDels were found in *T. qatarensis*–*T. simplex.* Plastome size is directly proportional to the number of SNPs and InDels. The large plastome had the most significant number of SNPs, InDels and vice versa. Similarly, in the same genus, the SNP and InDels were lower compared to subfamilies members. The genus and subfamily-based relation of SNPs and InDels was reported previously^83^. Mutational events occur in plastomes, including SNP, InDels, SSR repeats, and tandem inversions^84–86^. The association between SNP and InDels was previously reported^83,87,88^.

The IR regions of both *Tetraena* plastomes also lacked typically duplicated genes and the genes mentioned above. These genes, on the other hand, appeared in the SC regions. Four rRNA genes, for example, that are typically duplicated in plant plastomes, were found only in the SSC of *Tetraena* and *Zygophyllum* species plastomes. Similar results have previously been described in parasitic plants^62^. Unlike *Tetraena* and *Zygophyllum*, the plastomes of *L. tridentata*, *G. angustifolium*, and *Tr. terrestris* were far more extensive and included the often duplicated rRNA genes. The *rps19*, *rps12*, *ndhB*, *rps7*, and *rps8* genes were duplicated in *L. tridentata*, *G. angustifolium*, and *Tr. terrestris*; we found only one copy in all *Tetraena* and *Zygophyllum* species (Figs. 1, S1). As a result, the smaller number of genes in *Tetraena* and *Zygophyllum*'s IRs may have contributed to the genus's small plastome size. Recent studies of the number and distribution of repetitive sequences in fully sequenced rearranged plastid genomes such as legumes have revealed considerably more scattered repeats than in non-rearranged genomes^89^. Intron losses for the *rps12* and *clpP* genes have been found in the plastomes of *T. hamiensis* var. *qatarensis* and *T. simplex* and other angiosperm lineages. For example, both introns of the *clpP* gene have been deleted in Oleaceae, Onagraceae, Poaceae, and *Pinus*^90^.

In contrast, the intron in the 3′-end of *rps12* has been lost twice in the monocot order Asparagales^91^. However, the loss of introns from both *clpP* and *rps12* genes in *Teteraena* and *Zygophyllum* species is the first reported example of intron loss from both genes in the same plastid genome. We found that gene positions in IR boundaries have changed due to IR contraction in *T. hamiensis* var. *qatarensis* and *T. simplex* and related species, based on IR boundary studies. There is a slight variance near the IR boundary in both *T. hamiensis* var. *qatarensis* and *T. simplex* plastomes. The borders between the IRs and the SSC/LSC were significantly varied between *Tetraena* and other sampled species (Fig. 5). The shrinkage of the IR region in both *Tetraena* and *Zygophyllum* species was attributed to all these variations. Similar findings have already been observed in the Zygophyllaceae family^56,71^.

We used sliding window analysis and MISA to find highly variable regions and SSRs within the *T. hamiensis* var. *qatarensis* and *T. simplex* plastomes and compared them to 11 related plastomes. The sliding windows study revealed that, like in other taxa, the average mutation rate of the intergenic areas inside the SC regions was substantially more significant than that of the IR regions^92^. Due to lower selection pressures, higher mutation rates often result in more variety within intergenic spacers^93^. Like other functional repeat sequences, SSRs numbers differed in all these plastomes. The number of SSRs found varied from 161 (*T. mongolica*3) to 225 (*Z. fabago*1). There were 166 and 170 SSRs in the sequenced plastomes of *T. hamiensis* var. *qatarensis* and *T. simplex*, respectively (Fig. 3K) PolyA and polyT were the most prevalent mononucleotide repeats in *Tetraena* and *Zygophyllum*. *Lagerstroemia*^94^, *Primula*^95^, *Fritillaria*^96^, and *Allium*^96^ have all been shown to have polyA and polyT SSRs^97^.

The majority of Saudi Arabian *Zygophyllum* species were shifted to *Tetraena*, according to Beier et al.^34^’s most current taxonomic proposal of *Tetraena* Maxim. and *Zygophyllum*. Previously, researchers utilized several barcodes to accurately identify *Tetraena* and *Zygophyllum* species, including *rbcL*, *trnL-F*, and *matK.*^36,98,99^. However, to differentiate *Tetraena* and *Zygophyllum* species, we used chloroplast barcodes such as *matK*, *rbcL*, and *cssA* genes combined with 66 shared protein-coding genes. To know the evolutionary position of *T. hamiensis* var. *qatarensis* and *T. simplex*, phylogenetic analyses were performed using ML, NJ, MP, and BI based techniques. The trees generated by analyzing the whole plastomes (Figs. 6, S6), shared protein-coding genes (Figs. 7, S7), *matK* (Fig. S8), *rbcL* (Fig. S9), and *cssA* (Fig. S10) genes exhibited very identical topologies, indicating that *T. hamiensis* var. *qatarensis* and *T. simplex* are sister to the genus *Tetraena* and may not belong to *Zygophyllum*. Our findings support previous phylogenetic reports^36,100^ that *Z. simplex* is a sister to the genus *Tetraena*, not *Zygophyllum*, which is based on the sequence of *rbcl* morphological, anatomical attributes. They supported our recent findings that *Zygophyllum* is polyphyletic and may be subdivided into clusters^36,100^. Furthermore, the *Tetraena* species in our research were grouped into a single clade that was sister to the rest of the *Zygophyllum* species. *T. mongolica* formed a monophyletic group within the *Tetraena* genus. In contrast, *T. hamiensis* var. *qatarensis* was grouped with *T. simplex* into a single group based on complete genomes, proteins coding genes, and *matK* datasets. Similar findings have been reported in the others^36^. Phylogenetic relationships of 44 specimens representing seven species of Saudi Arabian *Tetraena* Maxim. and *Zygophyllum* L. were investigated using individual and combined chloroplast DNA data of *rbcL* and *trnL-F* by Alzahrani and Albokhari^101^ used individual and combined chloroplast DNA data of *rbcL* and *trnL-F* to study phylogenetic relationships of 44 specimens representing seven species of Saudi Arabian *Tetraena* Maxim. and *Zygophyllum* L. This is the first research to use super barcodes to investigate the evolutionary relationships of *Tetraena* and *Zygophyllum* species. Taxonomists have already conducted preliminary investigations to assess the possibility of plastomes in plant groups of closely related species. Bayly et al.^102^ provided a phylogenetic study on three taxa (*Eucalyptus*, *Corymbia*, and *Angophora*) and showed that the plastome might be used in lower-level genetic research. The plastome identifies species as organelle-scale "barcodes," according to Yang et al.^103^. The plastome, according to Zhang et al.^104^, might be considered a super-barcode for the closely related species. The ability of super-barcode was investigated in *Chrysanthemum* and *Ligularia*, respectively, by Xia et al.^105^ and Chen et al.^106^.

According to phylogenetic analysis and time divergence using beast2 the time divergence between Zygophyllaceae and Krameriaceae was calculated at 77.7 Ma (95 percent HPD: 77.2–78.0), i.e., Cretaceous, based on phylogenetic analysis and time divergence using beast2. The stem age of Zygophylloideae was 64.2 Ma (95% HPD: 57.2–72.6), whereas the crown age was 36.6 Ma (95% HPD: 32.9–43.8). Bell et al.^107^ published a similar conclusion, stating that the separation between Zygophyllaceae and Krameriaceae occurred about 70 Ma (49–88 Ma). In another study, the stem age of Zygophyllaceae at 60.9 Ma (34–90 Ma) by Magallón et al.^108^. However, our results are similar to a recent study where the stem age of Zygophyllaceae was set at 70 Ma (49–88 Ma)^107^ to infer the ages of Zygophylloideae and Asian *Zygophyllum*. Another study estimated the crown-group age of Zygophyllaceae to be 59.89 Ma (95% HPD: 38.14–80.95 Ma), which agrees with ages calculated using *rbcL* data having two secondary calibrations and ITS data having one secondary calibration^109^. Similarly, based on entire plastome (Fig. 6) and protein-coding genes (Fig. 7) datasets, the time divergence of *Zygophyllum* and *Tetraena* was 36.6 Ma and 34.4 Ma, respectively. *Zygophyllum* stem ages were 32.7 and 29.4 Ma, respectively, whereas *Tetraena* stem ages were 31.7 and 18.2 Ma based on full plastome and protein-coding genes. The stem age of Asian *Zygophyllum* was estimated to be 30.39 Ma (95% HPD: 21.53–39.81 Ma) in a previous study^110^. Both data based on the overall plastome dataset and protein-coding genes exhibited different conclusions in the instance of *T. simplex* and *T. hamiensis* var. *qatarensis* time divergence. *T. hamiensis* var. *qatarensis* and *T. simplex* separated from *T. mongolica* around 31.7 Ma based on the full plastome dataset, and 18.2 Ma based on protein-coding genes. *T. simplex* split 3.7 Ma from *T. hamiensis* var. *qatarensis* based on protein-coding genes data, whereas the divergence period was calculated to be 18.7 Ma based on entire plastome data. Wu et al.^110^ found similar results using the *rbcL* dataset, with a *T. simplex* divergence timing of 3.04 Ma. Our findings are consistent with previous findings, suggesting that *Zygophyllum* and *Tetraena* evolved separately, according to Bellstedt et al.^35^ and Wu et al.^110^.

## Conclusion

In the current study we sequenced and analyzed the plastomes of *T. hamiensis* var. *qatarensis* and *T. simplex*, revealing shorter length, shorter IR regions, and 16 deleted genes in SSC and IRs regions. Comparative analysis with other species showed genetic variations. Phylogenetic analysis supported current understanding of *Tetraena*'s status, with divergence times estimated at 36.6 Ma and 34.4 Ma for *Zygophyllum* and *Tetraena*, respectively. This dataset will provide a genetic resource for future research on *Tetraena*'s evolution, population genetics, and other biological functions.

## Methodology

The fresh juvenile leaves were collected from *T. hamiensis* var*. qatarensis* and *T. simplex* growing in their natural habitat Nizwa (22°46′22.7″N 57°27′56.8″E) were collected and transported in liquid nitrogen to the − 80 °C facility. The specimens were submitted to the University of Nizwa herbarium center under the voucher numbers UoN-TQ1 (*T. hamiensis* var*. qatarensis*) and UoN-TS1 (*T. simplex*). Saif Al-Hathmi one of the leading taxonomists at the Oman Botanical Garden, Muscat, Oman, identified the plants. The plant samples were collected and processed per the national guidelines and legislation. Hence, a permission permits (6210/10/73) was obtained from the Director-General of Nature Conservation, Ministry of Environment and Climate Affairs, Sultanate of Oman.

### DNA extraction and sequencing

The chloroplast (cp) DNA was isolated^111^. The genomic libraries were constructed according to the manufacturer’s protocol (Life Technologies USA, Eugene, OR, USA). Ion Shear™ Plus Reagents kit and Ion Xpress™ Plus, gDNA Fragment Library kit, were used to prepare (enzymatically) 400 bp fragments of the cp DNA. The prepared libraries were quantified using a Qubit 3.0 fluorometer followed by bioanalyzer (Agilent 2100 Bioanalyzer system, Life Technologies USA). The template was amplified using Ion OneTouch™ 2 and enriched using Ion OneTouch™ ES enrichment system Ion 530 & 520 OT2 reagents. Sequencing was performed using an Ion s5 sequencer (Life Technologies USA, Eugene, OR, USA). Samples were loaded on S5 530 chip according to the manufacturer’s protocol.

### Genome assembly and annotation

215,240,872 and 109,991,167 raw reads were obtained for *T. hamiensis* var*. qatarensis* and *T. simplex* plastomes, respectively. To eliminate the low-quality sequences, we screened the reads for a Phred score < 30. The plastomes were assembled using two methods to ensure the accuracy of plastomes assembly. GetOrganelle v 1.7.5 pipeline^112^ with SPAdes version 3.10.1 (http://bioinf.spbau.ru/spades) as assemblers were employed to de novo assemble the plastomes *T. hamiensis* var*. qatarensis* and *T. simplex*. Annotations were performed using CpGAVAS2^113^ and DOGMA (http://dogma.ccbb.utexas.edu/, China)^114^, whereas tRNAs can-SE (v.1.21)^115^ was used to detect the tRNA genes. Similarly, the start-stop codons, intron boundaries and manual alterations were performed by comparing the plastomes to reference genomes using Geneious Pro v.10.2.3.^116^ and tRNAs can-SE (v.1.21)^115^. The plastomes' structural features were illustrated using the chloroplot^117^ and circos^118^. Moreover, the genomic divergence was determined by mVISTA^119^ in shuffle-LAGAN mode using plastomes *T. hamiensis* var*. qatarensis* as reference.

### Repeat identification

We determined different functional repetitive sequences in *T. hamiensis* var. *qatarensis* and *T. simplex* in plastomes. Palindromic, forward, and reverse repeat sequences were determined using the REPuter^120^ online tool with 8 bp minimum repeat size conditions and 50 maximum computed repeats. Similarly, simple sequence repeats (SSRs) were calculated using MISA software^121^ using the conditions of ≥ 8 repeat units for one bp repeats; ≥ 6 repeat units for two bp repeats; ≥ 4 repeat units for 3 and 4 bp repeats and ≥ 3 repeat units for 5 and 6 bp repeats. Furthermore, tandem repeats were calculated using the online tool Tandem Repeats Finder v.4.09^122^.

### Genome divergence

The divergence in shared protein-coding genes and complete plastomes *T. hamiensis* var. *qatarensis, T. simplex*, and related species were determined. Comparative analysis was performed through multiple sequence alignment, and the gene order was compared and analyzed to refine the missing and dubious gene annotations. MAFFT version 7.222^123^ employed for plastomes annotations with default parameters. The pairwise sequence divergence was determined with Kimura’s two-parameter model (K2P)^123^. In DnaSP software v 6.13.03^124^ the sliding window analysis (200 bp window size and 100 bp step size) was used to calculate the relative synonyms codon usage (RSCU) value, variable sites and nucleotide variations (Pi).

### Phylogenetic analyses and divergence time

To determine the phylogenetic position of *T. hamiensis* var. *qatarensis* and *T. simplex* within the family Zygophyllaceae, 30 published plastome sequences of *Zygophyllum* species, three plastomes sequences of *Tetraena* species, *Larrea*, *Guaiacum* and *Tribulus* plastomes were downloaded from the NCBI database for phylogenetic analysis. Two *Krameria* species plastomes sequences were used as outgroup. Based on conserved gene order and structure, several alignments of whole plastomes were created^125^. The phylogenetic trees were generated using the four techniques listed below: MrBayes 3.1.2 was used for Bayesian inference; MEGA 6^126^ was used for maximum likelihood (ML) and neighbor-joining (NJ); and PAUP^127,128^ was used for maximum parsimony (MP). As per Akaike information criterion (AIC) for Bayesian posterior probabilities (PP) in BI analyses, the optimal substitution model GTR + G was examined using jModelTest version v2.1.02^129^. Starting with random trees and sampling 1 out of every 100 generations, the Markov Chain Monte Carlo (MCMC) technique was used to simulate 1,000,000 generations utilizing four incrementally heated chains. The values of the first 25% of trees were removed as burn-in for estimating the posterior probability. The maximum parsimony run was based on a heuristic search using the tree-bisection-reconnection (TBR) branch-swapping tree search criterion and 1000 random additions of sequence repetitions. Similarly, using a BIONJ tree^130^ as the starting tree and 1000 bootstrap replicates, the parameters for ML analysis were adjusted using the Kimura 2-parameter model with invariant sites and gamma-distributed rate heterogeneity.

A set of 58 shared genes, *rbcL* gene*, matK* gene, and *cssA* gene from the plastomes of the above species were aligned using MAFFT version 7.222^131^ under default parameters and by making various manual adjustments to preserve and improve reading frames in the second, third, fourth, and fifth tiers of phylogenies. As previously indicated and proposed by Asaf et al.^24^, the ML approach generated trees utilizing 58 shared genes, *matK* gene, *rbcL* gene, and *cssA* gene.

To conclude the divergence time of *Tetraena* with those of 30 *Zygophyllum* species, we used both entire plastomes and the concatenated data matrix. In BEAST^132^, a general time reversible (GTR + G) substitution model with four rate categories and a Yule tree speciation model with a lognormal relaxed clock model was utilized. We employed a fossil-based method to calibrate the molecular divergence by calculating an average substitution rate of 3.0 × 10^−9^ substitutions per site per year (s/s/y). The fossil record within Zygophyllaceae family is very sparse (reviewed by Bellstedt et al.^35^), no member of the present genera can be ascribed to the few reported fossils. Therefore, we employed a supplementary calibration strategy. We examined the data using concatenated protein-coding genes and entire plastomes to assess the robustness of our method. The *Zygophyllum* and *Tetraena* clades were specified as monophyletic, and we applied age constraints on four nodes with normal prior distributions^107,110^. To root the calibration time, we included two available outgroups species, *Krameria bicolor* and *Krameria lanceolata* from family Krameriaceae (the sister group to Zygophyllaceae). These outgroups were selected because they are all closely related to our research model species and have fossil records dating back to before the *Zygophyllum* genus^110^*.* Following the results of Bell et al.^107^ and Wu et al.^110^, the split between Zygophyllaceae and Krameriaceae was set at 70 Ma (49–88 Ma). Three separate MCMC runs of 50 million generations were used in the dating studies. The tree files from all three runs were combined with LOGCOMBINER. TRACER 1.5^133^ was used to test convergence and adequate sample sizes. We burned off 25% of the trees in each analysis. Finally, TREEANNOTATOR^134^ was used to construct the tree, and FIGTREE1.4 was used to display the tree with a 95% greatest posterior density (HPD).

## Supplementary Information

Supplementary Legends.

Supplementary Figure S1.

Supplementary Figure S2.

Supplementary Figure S3.

Supplementary Figure S4.

Supplementary Figure S5.

Supplementary Figure S6.

Supplementary Figure S7.

Supplementary Figure S8.

Supplementary Figure S9.

Supplementary Figure S10.

Supplementary Tables.

## Data Availability

All data generated or analyzed during this study are included in this published article. *T. hamiensis* var*. qatarensis* and *T. simplex* plastomes were submitted to NCBI with accession numbers (OM809718) and (OL943588) respectively.

## References

[1] Liu J (2018). Integrating a comprehensive DNA barcode reference library with a global map of yews (*Taxus* L.) for forensic identification. Mol. Ecol. Resour..

[2] Parveen I, Singh HK, Raghuvanshi S, Pradhan UC, Babbar SB (2012). DNA barcoding of endangered Indian Paphiopedilum species. Mol. Ecol. Resour..

[3] Li D-Z (2011). Comparative analysis of a large dataset indicates that internal transcribed spacer (ITS) should be incorporated into the core barcode for seed plants. Proc. Natl. Acad. Sci..

[4] Gueuning M (2019). Evaluating next-generation sequencing (NGS) methods for routine monitoring of wild bees: Metabarcoding, mitogenomics or NGS barcoding. Mol. Ecol. Resour..

[5] Gonzalez MA (2009). Identification of Amazonian trees with DNA barcodes. PLoS ONE.

[6] Chase MW, Reveal JL, Fay MF (2009). A subfamilial classification for the expanded asparagalean families Amaryllidaceae, Asparagaceae and Xanthorrhoeaceae. Bot. J. Linn. Soc..

[7] Hebert PD, Cywinska A, Ball SL, DeWaard JR (2003). Biological identifications through DNA barcodes. Proc. R. Soc. Lond. Ser. B Biol. Sci..

[8] Hollingsworth ML (2009). Selecting barcoding loci for plants: Evaluation of seven candidate loci with species-level sampling in three divergent groups of land plants. Mol. Ecol. Resour..

[9] Chase MW (2005). Land plants and DNA barcodes: Short-term and long-term goals. Philos. Trans. R. Soc. B Biol. Sci..

[10] Chen Q, Hu H, Zhang D (2022). DNA barcoding and phylogenomic analysis of the genus Fritillaria in China based on complete chloroplast genomes. Front. Plant Sci..

[11] Fu C-N (2019). Prevalence of isomeric plastomes and effectiveness of plastome super-barcodes in yews (Taxus) worldwide. Sci. Rep..

[12] Ji Y (2019). Testing and using complete plastomes and ribosomal DNA sequences as the next generation DNA barcodes in Panax (Araliaceae). Mol. Ecol. Resour..

[13] Gitzendanner MA, Soltis PS, Yi T-S, Li D-Z, Soltis DE (2018). Advances in Botanical Research.

[14] Li H-T (2019). Origin of angiosperms and the puzzle of the Jurassic gap. Nat. Plants.

[15] Nie Y (2019). Accounting for uncertainty in the evolutionary timescale of green plants through clock-partitioning and fossil calibration strategies. Syst. Biol..

[16] Asaf S (2021). The dynamic history of gymnosperm plastomes: Insights from structural characterization, comparative analysis, phylogenomics, and time divergence. Plant Genome.

[17] Chen Q, Wu X, Zhang D (2019). Phylogenetic analysis of *Fritillaria **cirrhosa* D. Don and its closely related species based on complete chloroplast genomes. PeerJ.

[18] Li X (2015). Plant DNA barcoding: From gene to genome. Biol. Rev..

[19] Coissac, E., Hollingsworth, P. M., Lavergne, S. & Taberlet, P. (Wiley Online Library, 2016). 10.1111/mec.13549 26821259

[20] Huang Y (2019). psbE-psbL and ndhA Intron, the promising plastid DNA barcode of fagopyrum. Int. J. Mol. Sci..

[21] Li X (2009). Study on conservation biology of Fritillaria cirrhosa.

[22] Huang H, Shi C, Liu Y, Mao S-Y, Gao L-Z (2014). Thirteen Camelliachloroplast genome sequences determined by high-throughput sequencing: Genome structure and phylogenetic relationships. BMC Evol. Biol..

[23] Guo H (2017). Complete chloroplast genome sequences of *Schisandra chinensis*: Genome structure, comparative analysis, and phylogenetic relationship of basal angiosperms. Sci. China Life Sci.

[24] Asaf S (2017). Chloroplast genomes of *Arabidopsis **halleri* ssp. *gemmifera* and *Arabidopsis **lyrata* ssp. *petraea*: Structures and comparative analysis. Sci. Rep..

[25] Khan A (2019). Complete chloroplast genomes of medicinally important Teucrium species and comparative analyses with related species from Lamiaceae. PeerJ.

[26] El Hadidi MN (1978). Adumbratio Florae Aethiopicae. 30: Zygophyllaceae. Webbia.

[27] Migahid A, Hammouda M (1996). Flora of Saudi Arabia, vol. I–III.

[28] Migahid, A. M. Flora of Saudi Arabia. (1978).

[29] Hosny A (1988). Genus *Zygophyllum* L. Arabia. Taeckholmia.

[30] Mandaville, J. P. *Flora of Eastern Saudi Arabia*. (Routledge, 2013).

[31] Chaudhary, S. *Flora of the Kingdom of Saudi Arabia (Vascular Plants)*. (National Agriculture and Water Research Center, National Herbarium, Ministry of Agriculture and Water, 2001).

[32] Soliman M, El-Tarras A, El-Awady M (2010). Seed exomorphic characters of some taxa from Saudi Arabia. J. Am. Sci..

[33] Waly NM, Al-Ghamdi FA, Al-Shamrani RI (2011). Developing methods for anatomical identification of the genus *Zygophyllum* L. (Zygophyllaceae) in Saudi Arabia. Life Sci. J..

[34] Beier B-A, Chase M, Thulin M (2003). Phylogenetic relationships and taxonomy of subfamily Zygophylloideae (Zygophyllaceae) based on molecular and morphological data. Plant Syst. Evol..

[35] Bellstedt D (2008). Phylogenetic relationships, character evolution and biogeography of southern African members of Zygophyllum (Zygophyllaceae) based on three plastid regions. Mol. Phylogenet. Evol..

[36] Sheahan MC, Chase MW (2000). Phylogenetic relationships within Zygophyllaceae based on DNA sequences of three plastid regions, with special emphasis on Zygophylloideae. Syst. Bot..

[37] Shaltout K, El-Halawany E, El-Garawany M (1997). Coastal lowland vegetation of eastern Saudi Arabia. Biodivers. Conserv..

[38] Batanouny, K. H. Ecology and Flora of Qatar. *Ecology and Flora of Qatar.* (1981).

[39] Collenette, S. *Illustrated guide to the flowers of Saudi Arabia*. (Scorpion, 1985).

[40] Collenette S (1999). The ceropegias of Saudi Arabia. British Cactus Succulent J..

[41] Mandaville JP (1986). Plant life in the Rub'al-Khali (the Empty Quarter), south-central Arabia. Proc. R. Soc. Edinb. Sect. B. Biol. Sci..

[42] Cornes M, Cornes CD (1989). wild flowering plants of Bahrain.

[43] Samuelsson G (1993). Inventory of plants used in traditional medicine in Somalia. IV. Plants of the families Passifloraceae-Zygophyllaceae. J. Ethnopharmacol..

[44] Wood JRI, Haig-Thomas H (1997). Handbook of the Yemen flora.

[45] Western AR (1989). The Flora of the United Arab Emirates: An Introduction.

[46] Hosny, A. I. *Revision of Genus Zygophyllum L., Sections Bipartia and Mediterranea in Egypt and Arabia*. (Unpublished, 1978).

[47] Böer B, Sargeant D (1998). Desert perennials as plant and soil indicators in Eastern Arabia. Plant Soil.

[48] Barth H-J (1999). Desertification in the eastern province of Saudi Arabia. J. Arid Environ..

[49] Sayed OH (1996). Adaptational responses of Zygophyllum qatarense Hadidi to stress conditions in a desert environment. J. Arid Environ..

[50] Karīm FM (2002). Wild Flowering Plants of the United Arab Emirates.

[51] Kisksi T, Guenaoui C, Fawzi N (2012). Early growth stages of the rare Acridocarpus orientalis in the UAE-A First step towards conservation. Nat. Resour..

[52] Laurent-Täckholm, V. & Drar, M. Students' flora of Egypt. (1956).

[53] Ghazanfar S, Osborne J (2015). Typification of *Zygophyllum*
*propinquum* Decne. and *Z.*
*coccineum* L. (Zygophyllaceae) and a key to Tetraena in SW Asia. Kew Bull..

[54] Zeng C-X, Zhang Y-X, Triplett JK, Yang J-B, Li D-Z (2010). Large multi-locus plastid phylogeny of the tribe Arundinarieae (Poaceae: Bambusoideae) reveals ten major lineages and low rate of molecular divergence. Mol. Phylogenet. Evol..

[55] Shapiro JA, von Sternberg R (2005). Why repetitive DNA is essential to genome function. Biol. Rev..

[56] Zhang L (2021). Comparative chloroplast genomics and phylogenetic analysis of Zygophyllum (Zygophyllaceae) of China. Front. Plant Sci..

[57] Asaf S, Ahmad W, Al-Harrasi A, Khan AL (2022). Uncovering the first complete plastome genomics, comparative analyses, and phylogenetic dispositions of endemic medicinal plant *Ziziphus **hajarensis* (Rhamnaceae). BMC Genomics.

[58] Lubna (2022). The plastome sequences of *Triticum **sphaerococcum* (ABD) and *Triticum turgidum* subsp. durum (AB) exhibit evolutionary changes, structural characterization, comparative analysis, phylogenomics and time divergence. Int. J. Mol. Sci..

[59] Mower JP, Vickrey TL (2018). Structural diversity among plastid genomes of land plants. Adv. Bot. Res..

[60] Tonti‐Filippini, J., Nevill, P. G., Dixon, K. & Small, I. Vol. 90, 808–818 (Wiley Online Library, 2017). 10.1111/tpj.13491 28112435

[61] Gruenstaeudl M, Nauheimer L, Borsch T (2017). Plastid genome structure and phylogenomics of Nymphaeales: Conserved gene order and new insights into relationships. Plant Syst. Evol..

[62] Delannoy E, Fujii S, Colas des Francs-Small C, Brundrett M, Small I (2011). Rampant gene loss in the underground orchid *Rhizanthella*
*gardneri* highlights evolutionary constraints on plastid genomes. Mol. Biol. Evol..

[63] Dobrogojski J, Adamiec M, Luciński R (2020). The chloroplast genome: A review. Acta Physiol. Plant..

[64] Li Y (2017). Gene losses and partial deletion of small single-copy regions of the chloroplast genomes of two hemiparasitic *Taxillus* species. Sci. Rep..

[65] Wolfe KH, Morden CW, Palmer JD (1992). Function and evolution of a minimal plastid genome from a nonphotosynthetic parasitic plant. Proc. Natl. Acad. Sci. USA.

[66] Park I (2019). Cuscuta species identification based on the morphology of reproductive organs and complete chloroplast genome sequences. Int. J. Mol. Sci..

[67] Braukmann TW, Kuzmina M, Stefanović S (2009). Loss of all plastid ndh genes in Gnetales and conifers: Extent and evolutionary significance for the seed plant phylogeny. Curr. Genet..

[68] Lubna (2021). The dynamic history of gymnosperm plastomes: Insights from structural characterization, comparative analysis, phylogenomics, and time divergence. Plant Genome.

[69] Lei W (2016). Intraspecific and heteroplasmic variations, gene losses and inversions in the chloroplast genome of *Astragalus **membranaceus*. Sci. Rep..

[70] Henriquez CL (2020). Complete chloroplast genomes of *Anthurium **huixtlense* and *Pothos** scandens* (Pothoideae, Araceae): Unique inverted repeat expansion and contraction affect rate of evolution. J. Mol. Evol..

[71] Wang X, Dorjee T, Chen Y, Gao F, Zhou Y (2022). The complete chloroplast genome sequencing analysis revealed an unusual IRs reduction in three species of subfamily Zygophylloideae. PLoS ONE.

[72] Zhang Y (2016). The complete chloroplast genome sequences of five Epimedium species: Lights into phylogenetic and taxonomic analyses. Front. Plant Sci..

[73] He L (2017). Complete chloroplast genome of medicinal plant *Lonicera japonica*: Genome rearrangement, intron gain and loss, and implications for phylogenetic studies. Molecules.

[74] Omelchenko DO (2020). Complete plastome sequencing of *Allium **paradoxum* reveals unusual rearrangements and the loss of the ndh genes as compared to *Allium **ursinum* and other onions. Gene.

[75] Yu J, Wang C, Gong X (2017). Degeneration of photosynthetic capacity in mixotrophic plants, *Chimaphila japonica* and *Pyrola **decorata* (Ericaceae). Plant Divers..

[76] Peredo EL, King UM, Les DH (2013). The plastid genome of *Najas*
*flexilis*: Adaptation to submersed environments is accompanied by the complete loss of the NDH complex in an aquatic angiosperm. PLoS ONE.

[77] Kim HT (2015). Seven new complete plastome sequences reveal rampant independent loss of the ndh gene family across orchids and associated instability of the inverted repeat/small single-copy region boundaries. PLoS ONE.

[78] Wu F-H (2009). Complete nucleotide sequence of *Dendrocalamus*
*latiflorus* and *Bambusa*
*oldhamii* chloroplast genomes. Tree Physiol..

[79] Ruhlman TA (2015). NDH expression marks major transitions in plant evolution and reveals coordinate intracellular gene loss. BMC Plant Biol..

[80] Dong WL (2018). Molecular evolution of chloroplast genomes of orchid species: Insights into phylogenetic relationship and adaptive evolution. Int. J. Mol. Sci..

[81] Chris Blazier J, Guisinger MM, Jansen RK (2011). Recent loss of plastid-encoded ndh genes within Erodium (Geraniaceae). Plant Mol. Biol..

[82] Sanderson MJ (2015). Exceptional reduction of the plastid genome of saguaro cactus (*Carnegiea gigantea*): Loss of the ndh gene suite and inverted repeat. Am. J. Bot..

[83] Abdullah, Henriquez CL, Croat TB, Poczai P, Ahmed I (2021). Mutational dynamics of aroid chloroplast genomes II. Front. Genet..

[84] Poczai P, Hyvönen J (2011). Identification and characterization of plastid trnF (GAA) pseudogenes in four species of Solanum (*Solanaceae*). Biotech. Lett..

[85] Jheng C-F (2012). The comparative chloroplast genomic analysis of photosynthetic orchids and developing DNA markers to distinguish Phalaenopsis orchids. Plant Sci..

[86] Shahzadi I (2019). Comparative analyses of chloroplast genomes among three Firmiana species: Identification of mutational hotspots and phylogenetic relationship with other species of Malvaceae. Plant Gene.

[87] Li J, Su Y, Wang T (2018). The repeat sequences and elevated substitution rates of the chloroplast accD gene in cupressophytes. Front. Plant Sci..

[88] Mes TH (2000). Hairpins involving both inverted and direct repeats are associated with homoplasious indels in non-coding chloroplast DNA of Taraxacum (Lactuceae: Asteraceae). Genome.

[89] Saski C (2005). Complete chloroplast genome sequence of *Glycine max* and comparative analyses with other legume genomes. Plant Mol. Biol..

[90] Jansen RK (2007). Analysis of 81 genes from 64 plastid genomes resolves relationships in angiosperms and identifies genome-scale evolutionary patterns. Proc. Natl. Acad. Sci..

[91] McPherson MA, Fay MF, Chase MW, Graham SW (2004). Parallel loss of a slowly evolving intron from two closely related families in Asparagales. Syst. Bot..

[92] Perry AS, Wolfe KH (2002). Nucleotide substitution rates in legume chloroplast DNA depend on the presence of the inverted repeat. J. Mol. Evol..

[93] Wang A, Yang M, Liu J (2005). Molecular phylogeny, recent radiation and evolution of gross morphology of the rhubarb genus Rheum (Polygonaceae) inferred from chloroplast DNA trn LF sequences. Ann. Bot..

[94] Zheng G (2020). Comparative analyses of chloroplast genomes from 13 Lagerstroemia (Lythraceae) species: Identification of highly divergent regions and inference of phylogenetic relationships. Plant Mol. Biol..

[95] Ren T, Yang Y, Zhou T, Liu Z-L (2018). Comparative plastid genomes of Primula species: Sequence divergence and phylogenetic relationships. Int. J. Mol. Sci..

[96] Bi Y (2018). Chloroplast genomic resources for phylogeny and DNA barcoding: A case study on Fritillaria. Sci. Rep..

[97] Xie D-F (2019). Phylogeny of Chinese Allium species in section Daghestanica and adaptive evolution of Allium (Amaryllidaceae, Allioideae) species revealed by the chloroplast complete genome. Front. Plant Sci..

[98] Bafeel SO (2011). Comparative evaluation of PCR success with universal primers of maturase K (matK) and ribulose-1, 5-bisphosphate carboxylase oxygenase large subunit (rbcL) for barcoding of some arid plants. Plant Omics.

[99] Maloukh L (2017). Discriminatory power of rbcL barcode locus for authentication of some of United Arab Emirates (UAE) native plants. 3 Biotech.

[100] Sheahan MC, Chase MW (1996). A phylogenetic analysis of Zygophyllaceae R. Br. based on morphological, anatomical and rbc L DNA sequence data. Bot. J. Linn. Soc..

[101] Alzahrani DA, Albokhari EJ (2017). Molecular phylogeny of Saudi Arabian *Tetraena* Maxim. and *Zygophyllum* L. (Zygophyllaceae) based on plastid DNA sequences. Bangladesh J. Plant Taxon..

[102] Bayly MJ (2013). Chloroplast genome analysis of Australian eucalypts—Eucalyptus, Corymbia, Angophora, Allosyncarpia and Stockwellia (Myrtaceae). Mol. Phylogenet. Evol..

[103] Yang J-B, Tang M, Li H-T, Zhang Z-R, Li D-Z (2013). Complete chloroplast genome of the genus *Cymbidium*: Lights into the species identification, phylogenetic implications and population genetic analyses. BMC Evol. Biol..

[104] Zhang Z, Zhang Y, Song M, Guan Y, Ma X (2019). Species identification of Dracaena using the complete chloroplast genome as a super-barcode. Front. Pharmacol..

[105] Xia Y (2016). The complete chloroplast genome sequence of *Chrysanthemum indicum*. Mitochondrial DNA Part A.

[106] Chen X (2018). Identification of Ligularia herbs using the complete chloroplast genome as a super-barcode. Front. Pharmacol..

[107] Bell CD, Soltis DE, Soltis PS (2010). The age and diversification of the angiosperms re-revisited. Am. J. Bot..

[108] Magallón S, Gómez-Acevedo S, Sánchez-Reyes LL, Hernández-Hernández T (2015). A metacalibrated time-tree documents the early rise of flowering plant phylogenetic diversity. New Phytol..

[109] Yu Y, Harris AJ, Blair C, He X (2015). RASP (Reconstruct Ancestral State in Phylogenies): A tool for historical biogeography. Mol. Phylogenet. Evol..

[110] Wu S-D (2015). Evolution of Asian interior arid-zone biota: Evidence from the diversification of Asian Zygophyllum (Zygophyllaceae). PLoS ONE.

[111] Shi C (2012). An improved chloroplast DNA extraction procedure for whole plastid genome sequencing. PLoS ONE.

[112] Jin J-J (2018). GetOrganelle: A simple and fast pipeline for de novo assembly of a complete circular chloroplast genome using genome skimming data. BioRxiv.

[113] Shi L (2019). CPGAVAS2, an integrated plastome sequence annotator and analyzer. Nucleic Acids Res..

[114] Wyman SK, Jansen RK, Boore JL (2004). Automatic annotation of organellar genomes with DOGMA. Bioinformatics.

[115] Schattner P, Brooks AN, Lowe TM (2005). The tRNAscan-SE, snoscan and snoGPS web servers for the detection of tRNAs and snoRNAs. Nucleic Acids Res..

[116] Kearse M (2012). Geneious Basic: An integrated and extendable desktop software platform for the organization and analysis of sequence data. Bioinformatics.

[117] Zheng S, Poczai P, Hyvönen J, Tang J, Amiryousefi A (2020). Chloroplot: An online program for the versatile plotting of organelle genomes. Front. Genet..

[118] Krzywinski M (2009). Circos: An information aesthetic for comparative genomics. Genome Res..

[119] Frazer KA, Pachter L, Poliakov A, Rubin EM, Dubchak I (2004). VISTA: Computational tools for comparative genomics. Nucleic Acids Res..

[120] Kurtz S (2001). REPuter: The manifold applications of repeat analysis on a genomic scale. Nucleic Acids Res..

[121] Beier S, Thiel T, Münch T, Scholz U, Mascher M (2017). MISA-web: A web server for microsatellite prediction. Bioinformatics.

[122] Benson G (1999). Tandem repeats finder: A program to analyze DNA sequences. Nucleic Acids Res..

[123] Katoh K, Toh H (2010). Parallelization of the MAFFT multiple sequence alignment program. Bioinformatics.

[124] Librado P, Rozas J (2009). DnaSP v5: A software for comprehensive analysis of DNA polymorphism data. Bioinformatics.

[125] Wicke S, Schneeweiss GM, Depamphilis CW, Müller KF, Quandt D (2011). The evolution of the plastid chromosome in land plants: Gene content, gene order, gene function. Plant Mol. Biol..

[126] Kumar S, Nei M, Dudley J, Tamura K (2008). MEGA: A biologist-centric software for evolutionary analysis of DNA and protein sequences. Brief. Bioinform..

[127] Asaf S (2016). Complete chloroplast genome of *Nicotiana **otophora* and its comparison with related species. Front. Plant Sci..

[128] Wu Z, Tembrock LR, Ge S (2015). Are differences in genomic data sets due to true biological variants or errors in genome assembly: An example from two chloroplast genomes. PLoS ONE.

[129] Posada D (2008). jModelTest: Phylogenetic model averaging. Mol. Biol. Evol..

[130] Gascuel O (1997). BIONJ: An improved version of the NJ algorithm based on a simple model of sequence data. Mol. Biol. Evol..

[131] Katoh K, Standley DM (2013). MAFFT multiple sequence alignment software version 7: Improvements in performance and usability. Mol. Biol. Evol..

[132] Suchard MA (2018). Bayesian phylogenetic and phylodynamic data integration using BEAST 1.10. Virus Evol..

[133] Rambaut A, Drummond AJ, Xie D, Baele G, Suchard MA (2018). Posterior summarization in Bayesian phylogenetics using Tracer 1.7. Syst. Biol..

[134] Helfrich, P., Rieb, E., Abrami, G., Lücking, A. & Mehler, A. In *Proceedings of the Eleventh International Conference on Language Resources and Evaluation (LREC 2018).*

