# Supplementary material for: Comparative plastome genomics, taxonomic delimitation and evolutionary divergences of Tetraena hamiensis var. qatarensis and Tetraena simplex (Zygophyllaceae)
Source: Sci Rep. 2023 May 8;13:7436. doi: 10.1038/s41598-023-34477-1 (PMC10167353; doi:10.1038/s41598-023-34477-1)
Supplement: Supplementary file 1 — Supplementary Legends. [file 41598_2023_34477_MOESM1_ESM.docx]

**Supplementary information**

**Table S1.** Gene composition in *Tetraena* plastome.

**Table S2.** Introns and exons lengths for the splitting genes in plastome of *T. hamiensis* var*. qatarensis* and *T. simplex*

**Table S3**. Average pairwise distance of plastome sequences from *T. hamiensis* var. *qatarensis* and *T. simplex* with related species.

**Table S4.** Average pairwise distance of plastome shared genes from *T. hamiensis* var. *qatarensis*, *T. simplex* and other related species.

**Table S5.** Codon usage in *T. hamiensis* var. *qatarensis* and *T. simplex* plastomes.

**Supplementary Figures**

**Figure S1.** Genome Map of the *T. hamiensis* var*. qatarensis* and *T. simplex*. Thick lines represent inverted repeat regions (IRs). IRs split the cp genome into single large copy (LSC) and single small copy (SSC) regions. The counterclockwise transcribing genes are drawn outside while the clockwise are inside the circle. Genes related to different functional groups are color-coded. The circle's dark and light green colors represent the GC and AC content.

**Figure S2.** Summary of genes lost across *T. hamiensis* var*. qatarensis*, *T. simplex* and *related* species plastomes. Blue shows the missing genes, whereas the red shows the genes duplicated in plastomes.

**Figure S3.** Sliding window analysis of nucleotide variability among the *T. hamiensis* var*. qatarensis*, *T. simplex,* and *related* plastomes (window length: 200 bp; step size: 100 bp), (A) Nucleotide variability among *Tetraena* plastomes (B) Nucleotide variability among *Zygophyllum* plastomes (C) Nucleotide variability among all thirteen plastomes.

**Figure S4.** Visual alignment of *T. hamiensis* var*. qatarensis*, *T. simplex,* and *related* plastomes. VISTA-based identity plot showing sequence identities among thirteen plastomes, using *T. hamiensis* var*. qatarensis* as a reference. Genome regions are color-coded as protein-coding, rRNA coding, tRNA coding, or conserved noncoding sequences (CNS). The x-axis represents the coordinate in the plastome. Annotated genes are displayed along the top. The sequence similarity of the aligned regions is shown as horizontal bars indicating the average percent identity between 50% and 100%.

**Figure S5.** Comparisons of border distances between adjacent genes and junctions of LSC, SSC, and two IR regions among *T. hamiensis* var*. qatarensis*, *T. simplex,* and *related* plastomes. Boxes above or below the central line indicate the adjacent border genes. The figure does not scale with sequence length and only shows relative changes at or near the IR/SC borders.

**Figure S6.** Complete cp genome-based phylogenetic tree of *T. hamiensis* var*. qatarensis* and *T. simplex*. The entire genome dataset was analyzed using the maximum likelihood (ML) method. Numbers above the branches represent bootstrap values in the (ML, MP, and NJ trees. Different colors represent subgroupings in the Zygophyllaceae family.

**Figure S7.** The phylogenetic tree is based on 58 shared protein-coding genes of *T. hamiensis* var*. qatarensis*, *T. simplex,* and *related* plastomes using the maximum likelihood (ML) method. Numbers above the branches represent bootstrap values in the (ML, MP, and NJ trees. Different colors represent subgroupings in the Zygophyllaceae family.

**Figure S8.** Phylogenetic analysis (ML) using *matK* gene from 40 species with available plastome sequences in the Zygophyllaceae. The numbers above the n​o​d​e​s​ represent ML bootstrap values.

**Figure S9.** Phylogenetic analysis (ML) using the *rbcL* gene from 40 species with available plastome sequences in the Zygophyllaceae. Numbers above to the n​o​d​e​s​ represent ML bootstrap values.

**Figure S10.** Phylogenetic analysis (ML) using *cssA* gene from 40 species with available plastome sequences in the Zygophyllaceae. The numbers above the n​o​d​e​s​ represent ML bootstrap values.
