# Supplementary figures and images for: Comparative plastome genomics, taxonomic delimitation and evolutionary divergences of Tetraena hamiensis var. qatarensis and Tetraena simplex (Zygophyllaceae)
Source: Sci Rep. 2023 May 8;13:7436. doi: 10.1038/s41598-023-34477-1 (PMC10167353; doi:10.1038/s41598-023-34477-1)

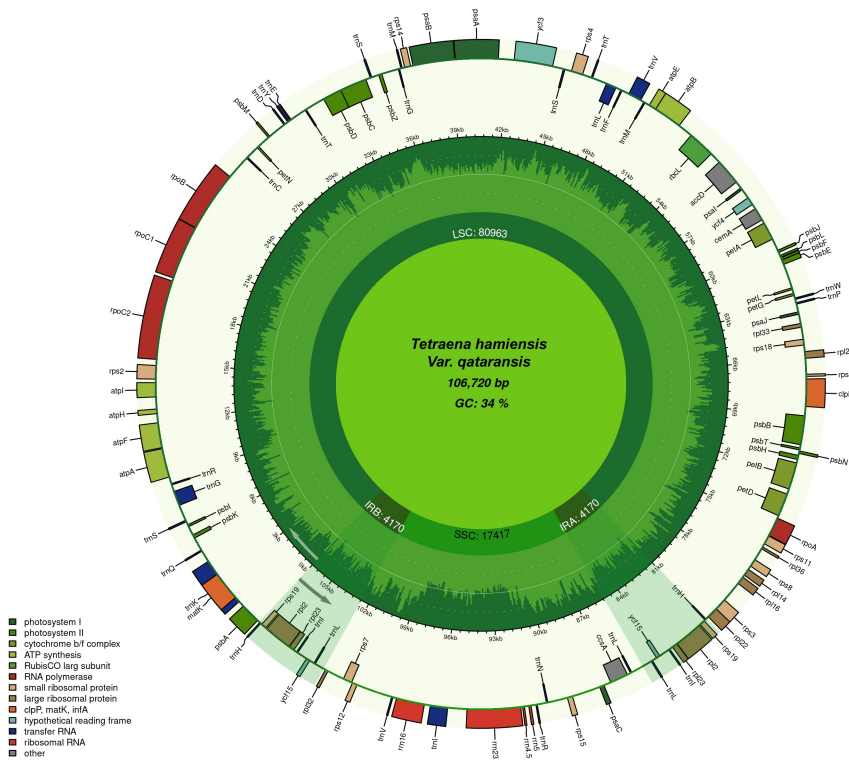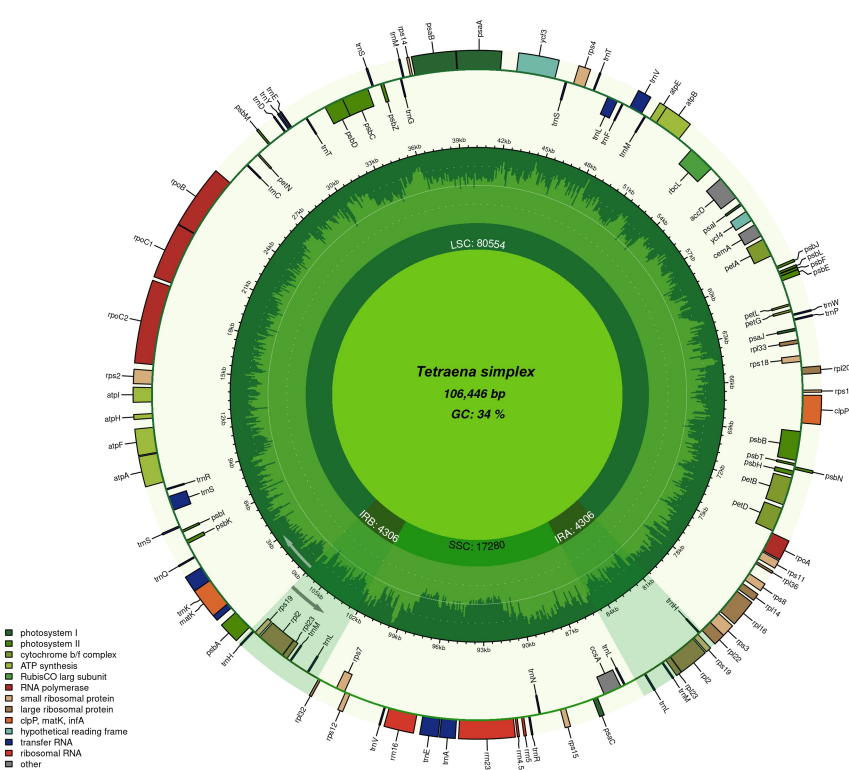

Supplement: Supplementary file 2 — Supplementary Figure S1. [file 41598_2023_34477_MOESM2_ESM.pdf]

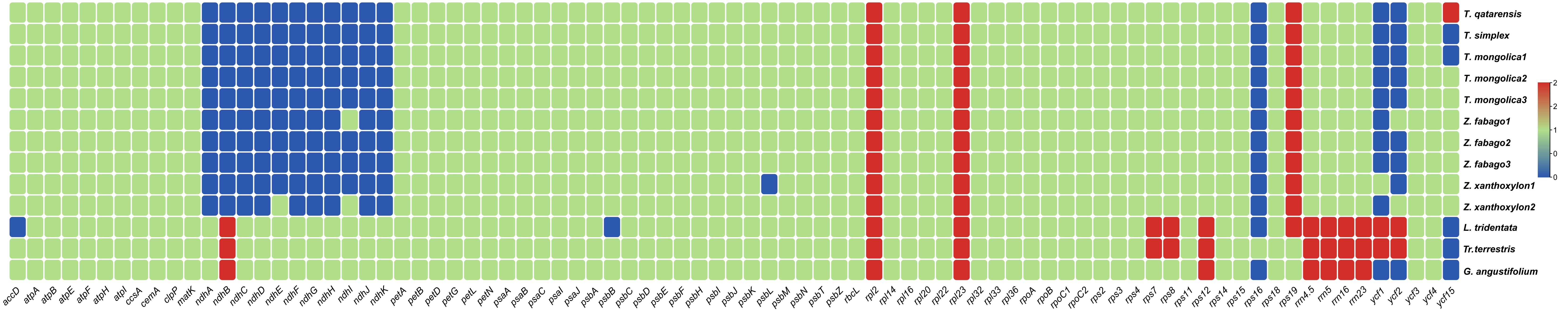

Supplement: Supplementary file 3 — Supplementary Figure S2. [file 41598_2023_34477_MOESM3_ESM.pdf]

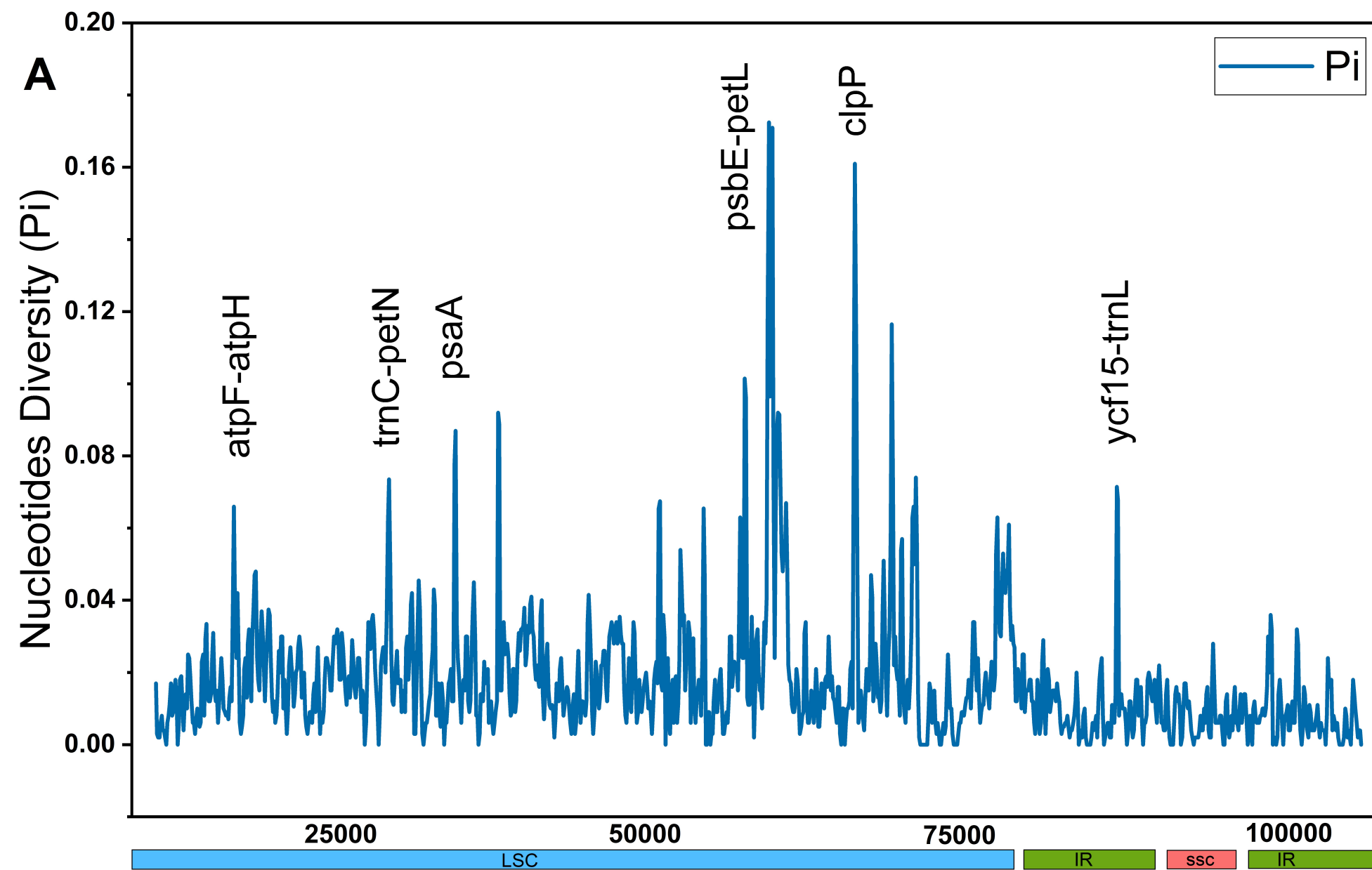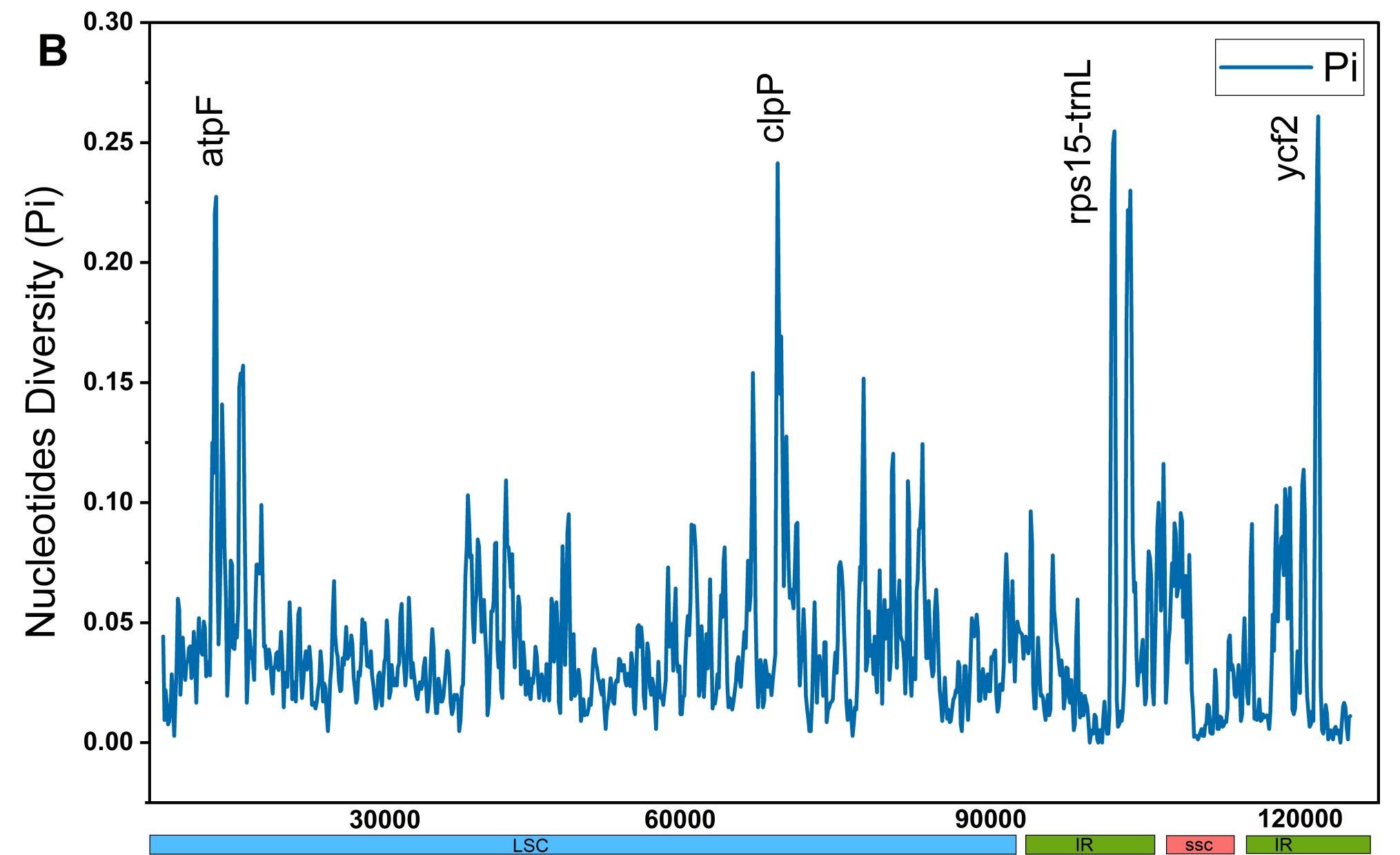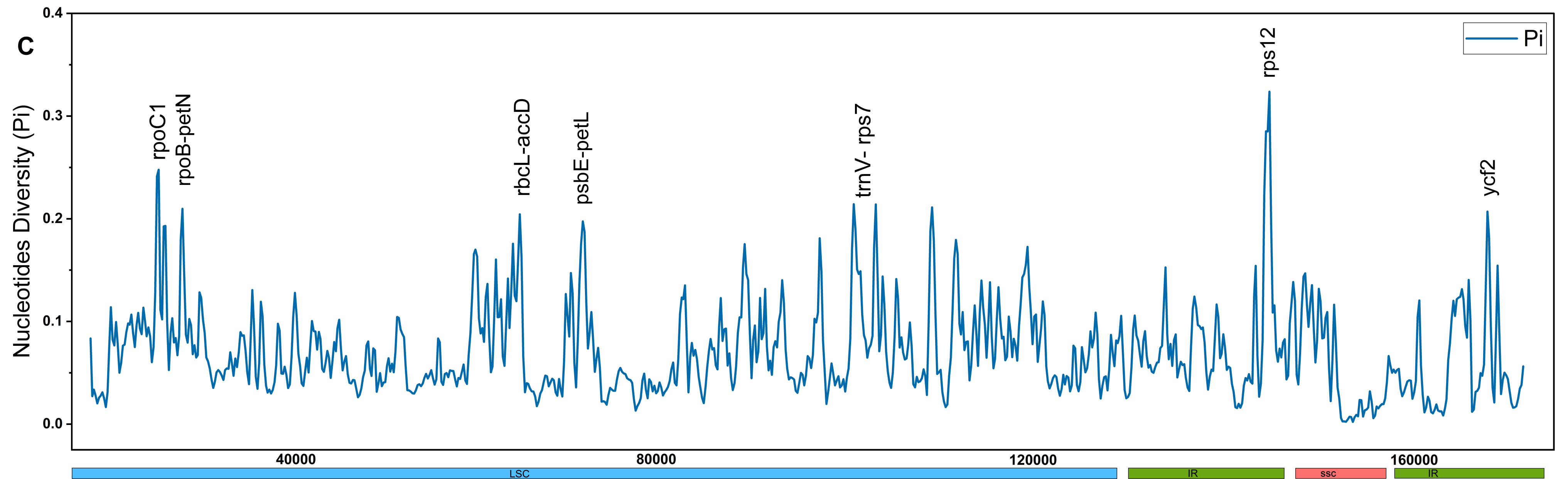

Supplement: Supplementary file 4 — Supplementary Figure S3. [file 41598_2023_34477_MOESM4_ESM.pdf]

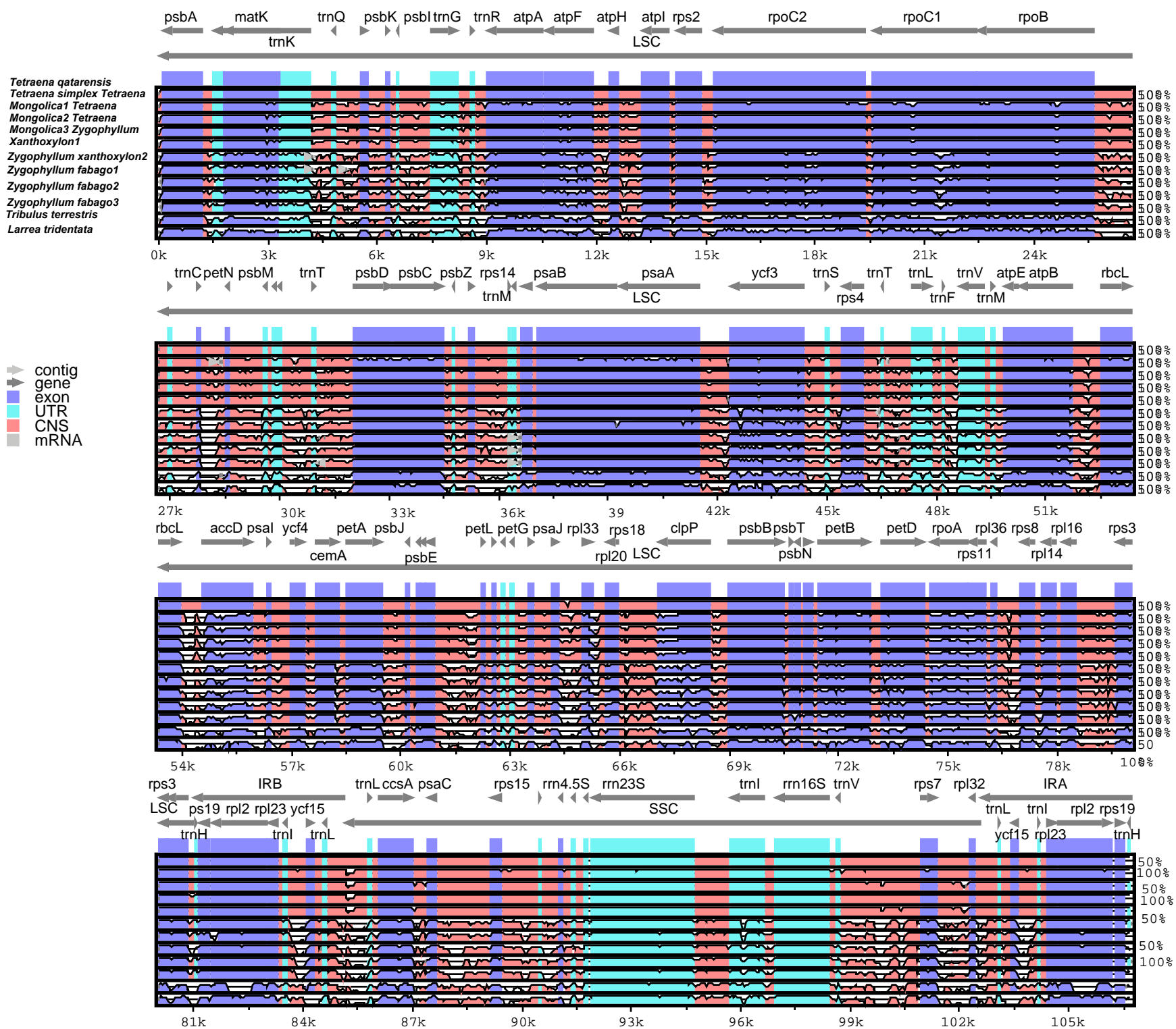

Supplement: Supplementary file 5 — Supplementary Figure S4. [file 41598_2023_34477_MOESM5_ESM.pdf]

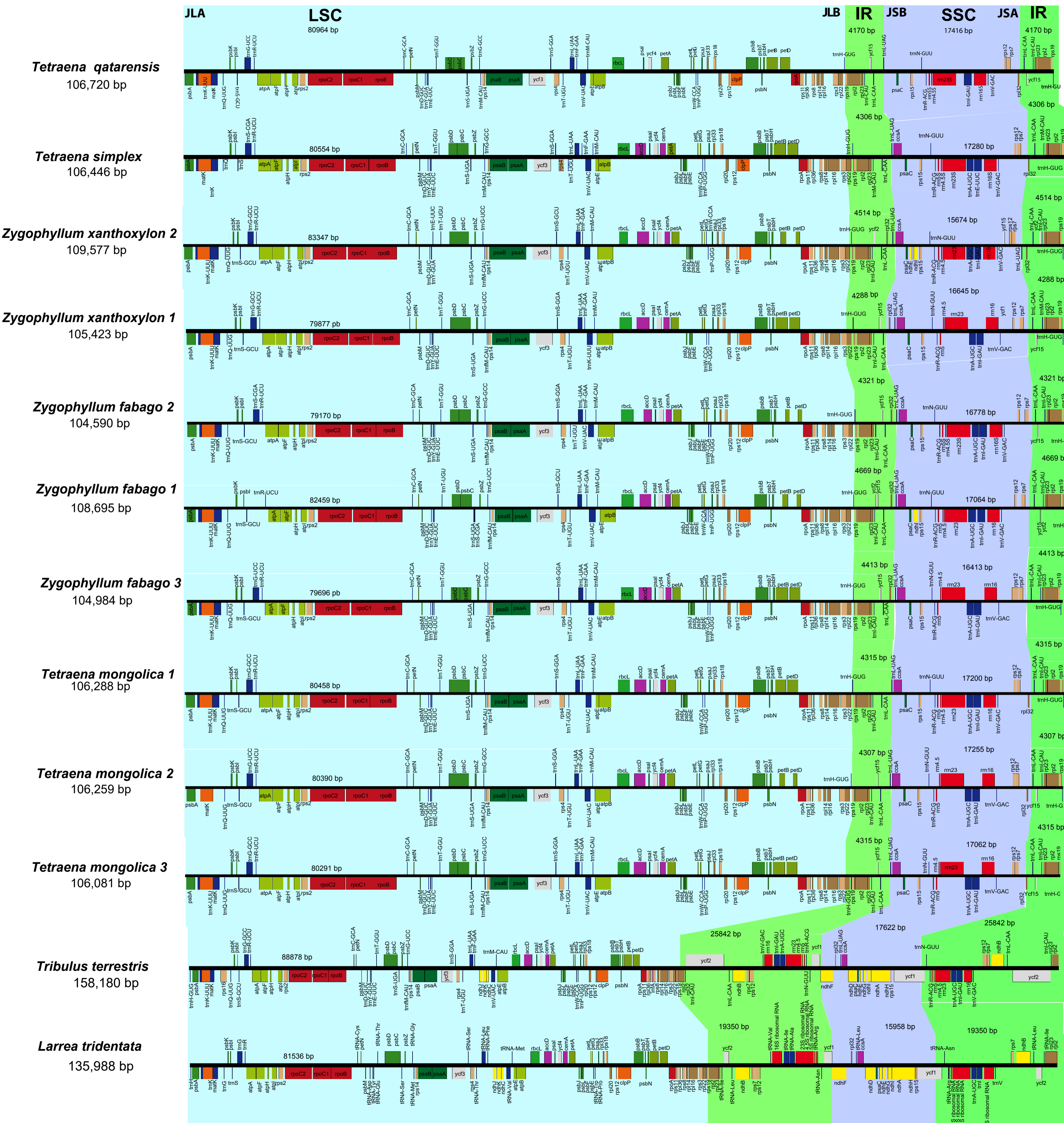

Supplement: Supplementary file 6 — Supplementary Figure S5. [file 41598_2023_34477_MOESM6_ESM.pdf]

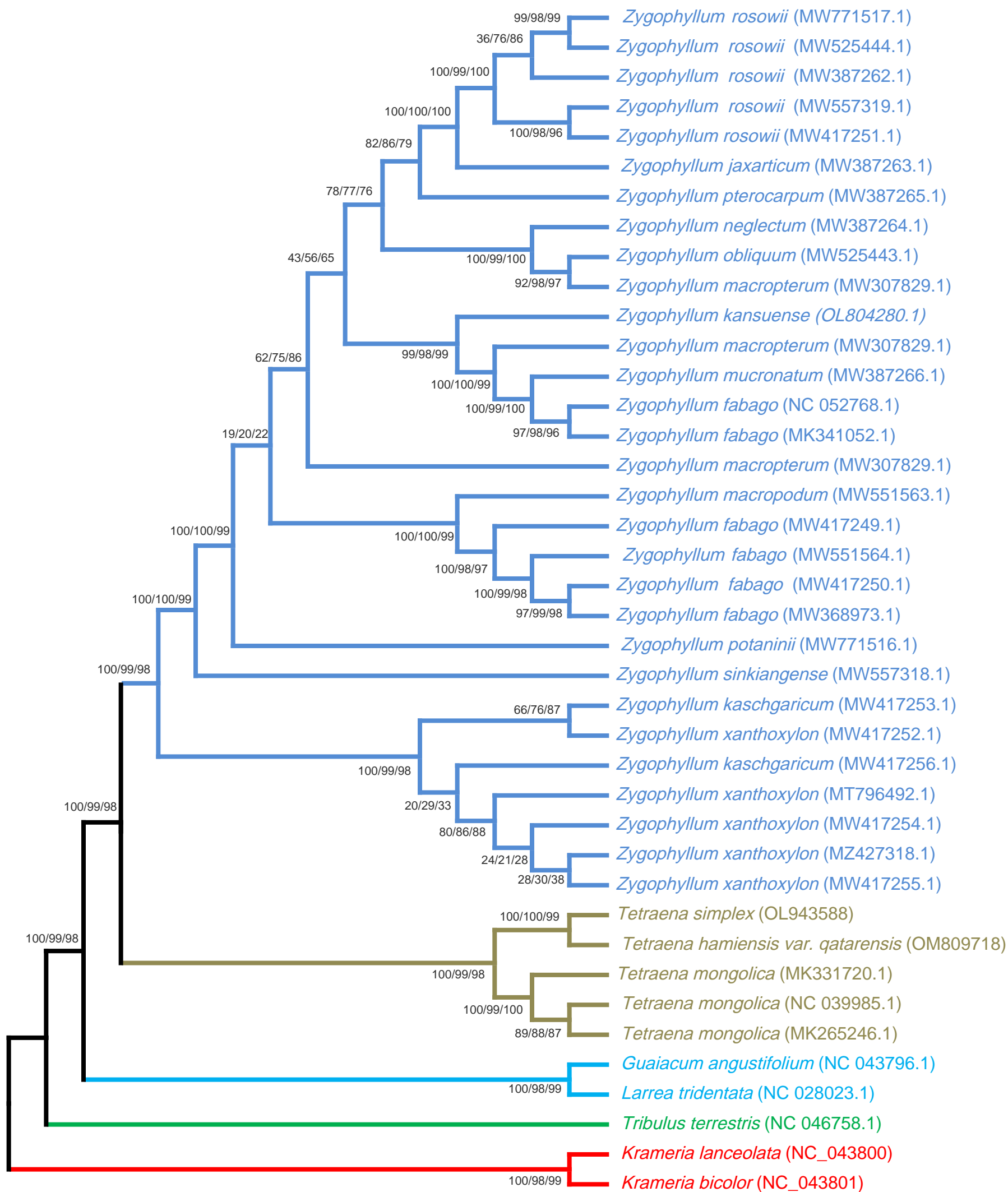

Supplement: Supplementary file 7 — Supplementary Figure S6. [file 41598_2023_34477_MOESM7_ESM.pdf]

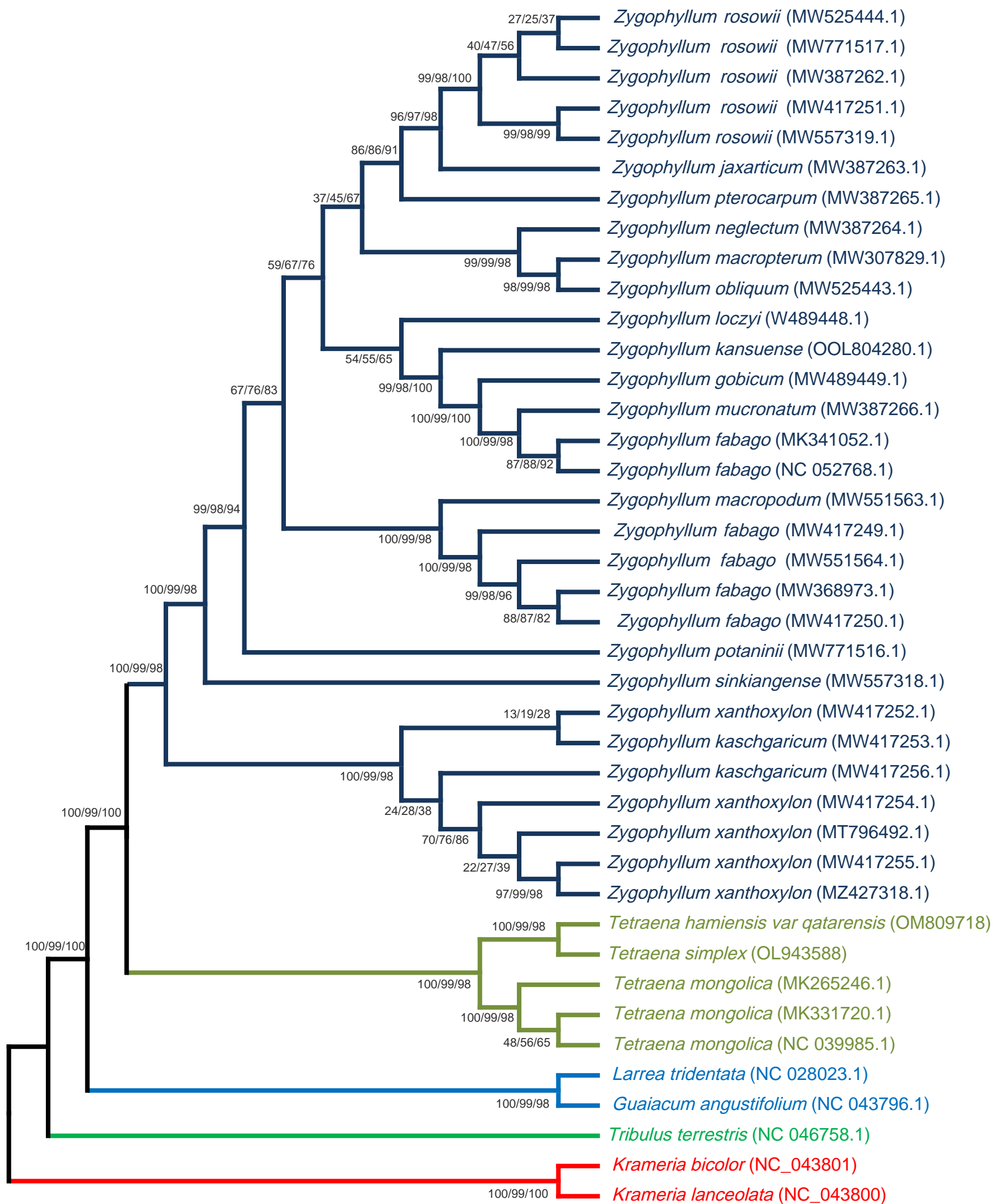

Supplement: Supplementary file 8 — Supplementary Figure S7. [file 41598_2023_34477_MOESM8_ESM.pdf]

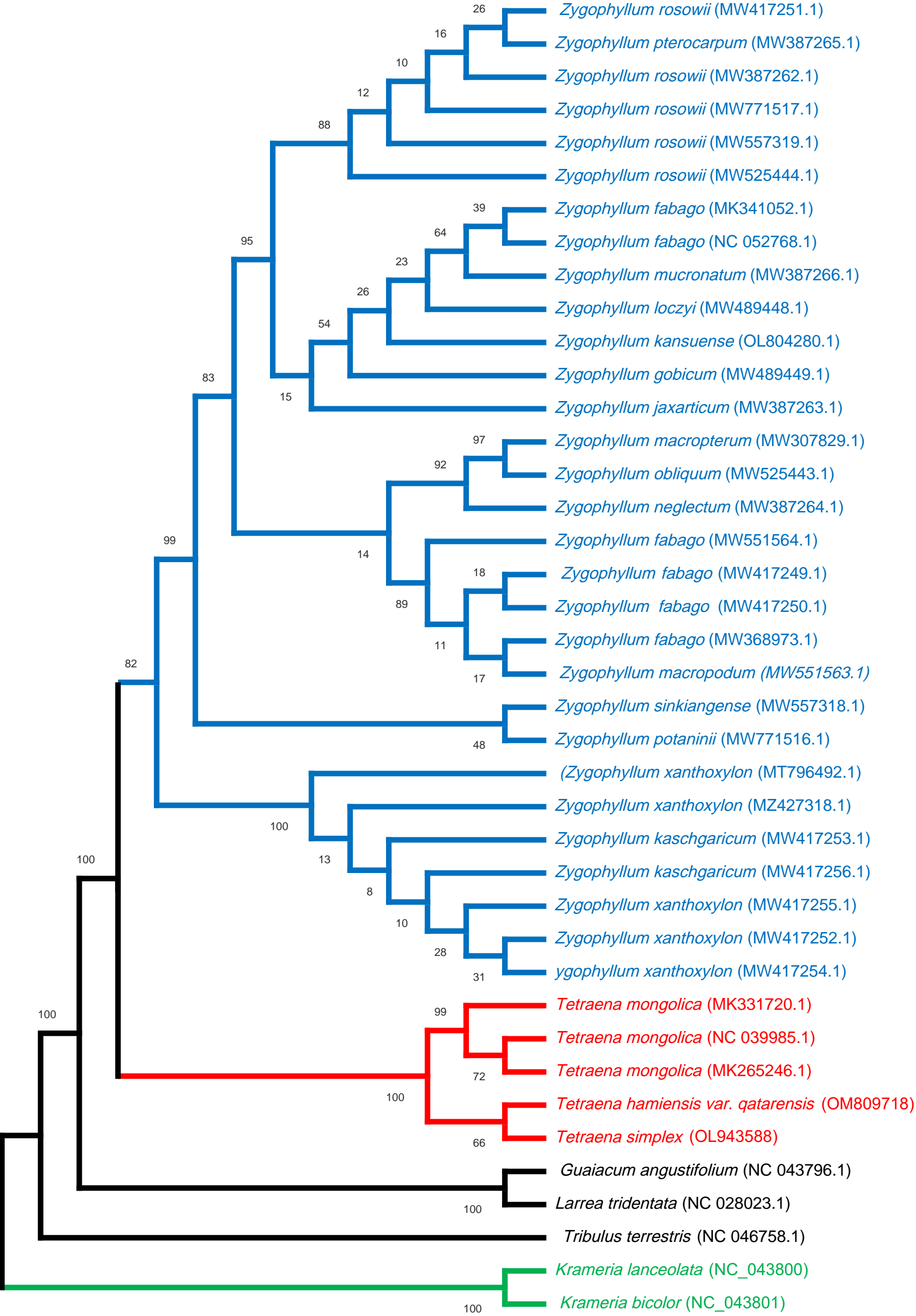

Supplement: Supplementary file 9 — Supplementary Figure S8. [file 41598_2023_34477_MOESM9_ESM.pdf]

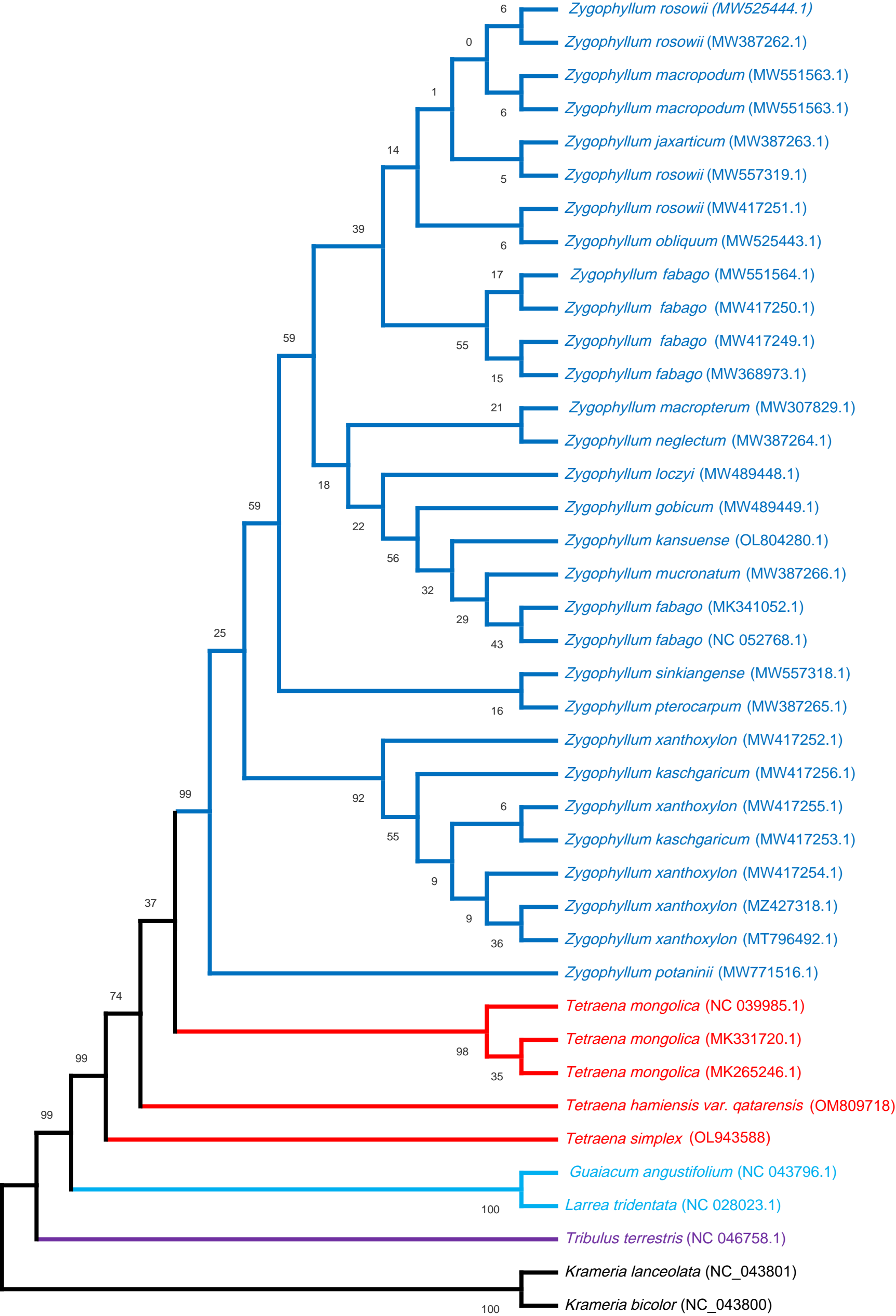

Supplement: Supplementary file 10 — Supplementary Figure S9. [file 41598_2023_34477_MOESM10_ESM.pdf]

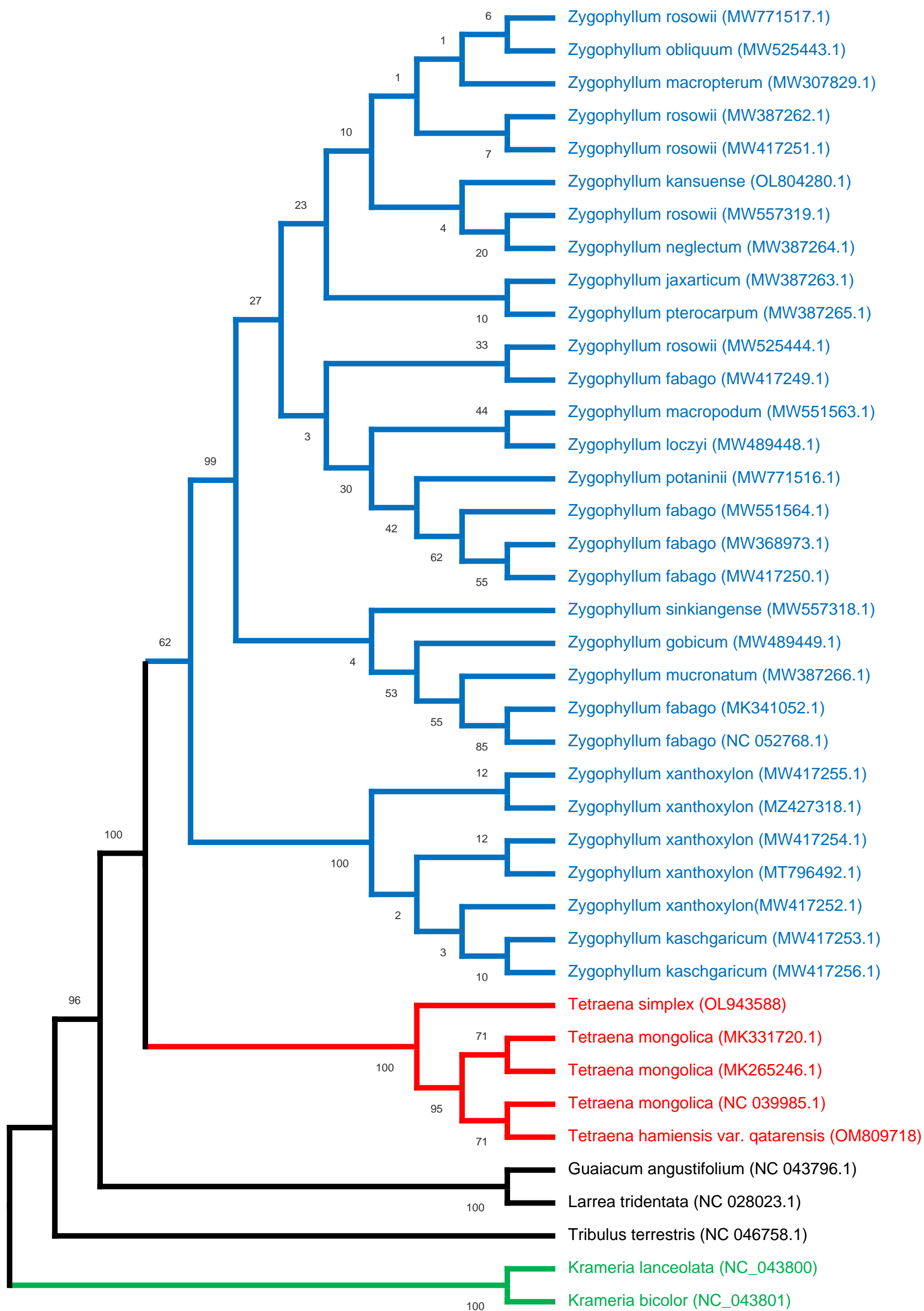

Supplement: Supplementary file 11 — Supplementary Figure S10. [file 41598_2023_34477_MOESM11_ESM.pdf]
