# Supplementary material for: Comparative plastome genomics, taxonomic delimitation and evolutionary divergences of Tetraena hamiensis var. qatarensis and Tetraena simplex (Zygophyllaceae)
Source: Sci Rep. 2023 May 8;13:7436. doi: 10.1038/s41598-023-34477-1 (PMC10167353; doi:10.1038/s41598-023-34477-1)
Supplement: Supplementary file 12 — Supplementary Tables. [file 41598_2023_34477_MOESM12_ESM.docx]

**Table S1.** Gene composition in *Tetraena* plastome.

| Category of genes | Group of genes | Name of genes |
| --- | --- | --- |
| Genes for photosynthesis | Subunits of ATP synthase | *atpA, atpB, atpE, atpF, atpH, atpI* |
| Genes for photosynthesis | Subunits of photosystem II | *psbA, psbB, psbC, psbD, psbE, psbF, psbI, psbJ, psbK, psbL, psbN, psbT, psbZ, ycf3* |
| Genes for photosynthesis | Subunits of cytochrome b/f complex | *petA, petB, petD, petG, petN* |
| Genes for photosynthesis | Subunits of photosystem I | *psaA, psaB, psaC, psaI, psaJ* |
| Genes for photosynthesis | Subunit of rubisco | *rbcL* |
|  |  |  |
| Self-replication | Large subunit of ribosome | *rpl14, rpl2*, rpl22, rpl23, rpl23, rpl33, rpl36* |
| Self-replication | DNA dependent RNA polymerase | *rpoA, rpoB, rpoC2* |
| Self-replication | Small subunit of ribosome | *rps11, rps12, rps14, rps18, rps19, rps19, rps2, rps3, rps4, rps7, rps8* |
|  |  |  |
| Other genes | Subunit of Acetyl-CoA-carboxylase | *accD* |
| Other genes | c-type cytochrom synthesis gene | *ccsA* |
| Other genes | Envelop membrane protein | *cemA* |
| Other genes | Protease | *clpP* |
| Other genes | Maturase | *matK* |
|  |  |  |
| Unkown | Conserved open reading frames | *ycf4* |

*****Genes are duplicated

**Table S2.** Introns and exons lengths for the splitting genes in plastomes of *T. hamiensis* var*. qatarensis* and *T. simplex*

| Genes | Start |  | End |  | ExonI |  | IntronI |  | ExonII |  | IntronII |  | ExonIII |  |
| --- | --- | --- | --- | --- | --- | --- | --- | --- | --- | --- | --- | --- | --- | --- |
|  | **TQ** | **TS** | **TQ** | **TS** | **TQ** | **TS** | **TQ** | **TS** | **TQ** | **TS** | **TQ** | **TS** | **TQ** | **TS** |
| *atpF* | 10585 | 10593 | 11898 | 11906 | 145 | 145 | 759 | 759 | 410 | 410 |  |  |  |  |
| *clpP* | 67036 | 66850 | 68404 | 68293 | 69 | 71 | 843 | 856 | 525 | 517 |  |  |  |  |
| *petB* | 71459 | 71212 | 72879 | 72671 | 6 | 6 | 773 | 810 | 642 | 642 |  |  |  |  |
| *petD* | 73161 | 72872 | 74375 | 74135 | 8 | 8 | 732 | 781 | 475 | 475 |  |  |  |  |
| *rpl2* | 81492 | 81173 | 82987 | 82671 | 394 | 394 | 629 | 632 | 473 | 473 |  |  |  |  |
| *rpoC1* | 19560 | 19527 | 22409 | 22356 | 456 | 432 | 792 | 793 | 1602 | 1605 |  |  |  |  |
| *ycf3* | 42344 | 42263 | 44378 | 44288 | 124 | 124 | 767 | 767 | 230 | 230 | 761 | 752 | 153 | 153 |
| *rps12* | 66790 | 66585 | 100874 | 100528 | 114 | 114 |  |  | 258 | 258 |  |  |  |  |
| *trnK-UUU* | 1490 | 1508 | 4140 | 4142 | 37 | 37 | 2579 | 2563 | 35 | 35 |  |  |  |  |
| *trnE-UUC* | 95718 | 95373 | 96634 | 96288 | 32 | 32 | 845 | 844 | 40 | 40 |  |  |  |  |
| *trnA-UGC* |  | 94522 |  | 95306 |  | 37 |  | 692 |  | 56 |  |  |  |  |
| *trnS-CGA* | 7437 | 7447 | 8210 | 8208 | 31 | 31 | 683 | 671 | 60 | 60 |  |  |  |  |
| *trnV-UAC* | 48619 | 48474 | 49318 | 49168 | 36 | 36 | 608 | 603 | 56 | 56 |  |  |  |  |
| *trnL-UAA* | 47336 | 47211 | 47878 | 47757 | 35 | 35 | 458 | 462 | 50 | 50 |  |  |  |  |

**Table S3**. Average pairwise distance of plastome sequences from *T. hamiensis* var. *qatarensis* and *T. simplex* with related species.

| *T. qatarensis* |  |  |  |  |  |  |  |  |  |  |  |
| --- | --- | --- | --- | --- | --- | --- | --- | --- | --- | --- | --- |
| *T. simplex* | 0.027072 |  |  |  |  |  |  |  |  |  |  |
| *T. mongolica1* | 0.023248 | 0.028160 |  |  |  |  |  |  |  |  |  |
| *T. mongolica2* | 0.023064 | 0.027980 | 0.000349 |  |  |  |  |  |  |  |  |
| *T. mongolica3* | 0.023089 | 0.028186 | 0.000434 | 0.000453 |  |  |  |  |  |  |  |
| *Z. fabago1* | 0.070781 | 0.076166 | 0.059267 | 0.059280 | 0.058934 |  |  |  |  |  |  |
| *Z. fabago2* | 0.070100 | 0.075840 | 0.058822 | 0.058867 | 0.058511 | 0.008800 |  |  |  |  |  |
| *Z. fabago3* | 0.070078 | 0.075589 | 0.058689 | 0.058651 | 0.058373 | 0.000488 | 0.008762 |  |  |  |  |
| *Z. xanthoxylon1* | 0.068762 | 0.074726 | 0.057292 | 0.057333 | 0.056971 | 0.028919 | 0.027896 | 0.028358 |  |  |  |
| *Z. xanthoxylon2* | 0.069398 | 0.075351 | 0.057963 | 0.057992 | 0.057643 | 0.029222 | 0.027975 | 0.028456 | 0.000526 |  |  |
| *Tr. terrestris* | 0.164381 | 0.163262 | 0.153358 | 0.153229 | 0.153276 | 0.143593 | 0.140574 | 0.141656 | 0.142769 | 0.147304 |  |
| *L. tridentata* | 0.177183 | 0.178031 | 0.167548 | 0.167521 | 0.167477 | 0.159984 | 0.157614 | 0.158615 | 0.157962 | 0.162484 | 0.134426 |

**Table S4.** Average pairwise distance of plastome shared genes from *T. hamiensis* var. *qatarensis*, *T. simplex* and other related species.

|  | *T. qatarensis* | *T. simplex* | *T. mongolica1* | *T. mongolica2* | *T. mongolica3* | *Z. fabago1* | *Z. fabago2* | *Z. fabago3* | *Z. xanthoxylon1* | *Z. xanthoxylon2* | *Tr. terrestris* | *L. tridentata* |
| --- | --- | --- | --- | --- | --- | --- | --- | --- | --- | --- | --- | --- |
| *atpA* |  | 0.04056442 | 0.01527847 | 0.01594263 | 0.01527298 | 0.04128478 | 0.04340912 | 0.04128478 | 0.04679428 | 0.04679428 | 0.08810284 | 0.10203391 |
| *atpB* |  | 0.0204409035 | 0.0224985595 | 0.0224985595 | 0.0224985595 | 0.0494263986 | 0.0516140261 | 0.0494263986 | 0.0436673040 | 0.0436673040 | 0.0824027878 | 0.1045537450 |
| *atpE* |  | 0.0000000000 | 0.0231992591 | 0.0231992591 | 0.0231992591 | 0.0536491342 | 0.0509062306 | 0.0536491342 | 0.0451136943 | 0.0451136943 | 0.1456405560 | 0.1152330859 |
| *atpF* |  | 0.029552062 | 0.033579421 | 0.033527058 | 0.033527058 | 0.059637782 | 0.061260352 | 0.060470995 | 0.058983128 | 0.059080918 | 0.168599577 | 0.142457194 |
| *atpH* |  | 0.0419503409 | 0.0000000000 | 0.0000000000 | 0.0000000000 | 0.0206632087 | 0.0206632087 | 0.0206632087 | 0.0207197237 | 0.0207197237 | 0.0645197082 | 0.0463591107 |
| *atpI* |  | 0.001345215 | 0.010835211 | 0.010835211 | 0.010835211 | 0.027446603 | 0.024599578 | 0.027446603 | 0.028842871 | 0.028842871 | 0.078733401 | 0.070777055 |
| *ccsA* |  | 0.0189000426 | 0.0010365453 | 0.0000000000 | 0.0010365453 | 0.0395028490 | 0.0395322177 | 0.0395028490 | 0.0384354867 | 0.0384354867 | 0.1381644574 | 0.1194589582 |
| *clpP* |  | 0.0654805807 | 0.0681392190 | 0.0672766801 | 0.0681392190 | 0.1708563561 | 0.1778689390 | 0.1722702219 | 0.1520427241 | 0.1520427241 | 0.6426484026 | 0.5050782084 |
| *matk* |  | 0.0220551277 | 0.0281939520 | 0.0281939520 | 0.0275039416 | 0.0618333766 | 0.0604192213 | 0.0618333766 | 0.0619253452 | 0.0619253452 | 0.1985384957 | 0.1813951095 |
| *petA* |  | 0.0160571234 | 0.0258486962 | 0.0258486962 | 0.0269563533 | 0.0470263214 | 0.0402569452 | 0.0470263214 | 0.0414416809 | 0.0414416809 | 0.1035185138 | 0.0840460250 |
| *petB* |  | 0.0169585157 | 0.0062277373 | 0.0024572289 | 0.0069213571 | 0.0116656221 | 0.0119032544 | 0.0336828111 | 0.0331591580 | 0.0125693829 | 0.0729086195 | 0.0666497108 |
| *petD* |  | 0.0070022465 | 0.0059330123 | 0.0016423200 | 0.0055795657 | 0.0080121434 | 0.0092114116 | 0.0186707824 | 0.0145015952 | 0.0071999263 | 0.0483366774 | 0.0605567825 |
| *PetG* |  | 0.0000000000 | 0.0088780712 | 0.0088780712 | 0.0088780712 | 0.0459435095 | 0.0459435095 | 0.0459435095 | 0.0459435095 | 0.0459435095 | 0.0467965085 | 0.0562855413 |
| *petL* |  | 0.0000000000 | 0.0105620589 | 0.0105620589 | 0.0105620589 | 0.0318321718 | 0.0318321718 | 0.0318321718 | 0.0210950300 | 0.0210950300 | 0.1147137259 | 0.1016770836 |
| *petN* |  | 0.0000000000 | 0.0000000000 | 0.0000000000 | 0.0000000000 | 0.0211995709 | 0.0226394226 | 0.0205462111 | 0.0226394226 | 0.0211995709 | 0.0580485022 | 0.0829403686 |
| *psaA* |  | 0.0391037913 | 0.0027017588 | 0.0027017588 | 0.0029727738 | 0.0123584362 | 0.0115148848 | 0.0123584362 | 0.0120782027 | 0.0121002182 | 0.0410615570 | 0.0333217840 |
| *psaB* |  | 0.0364269711 | 0.0073002894 | 0.0068399688 | 0.0068399688 | 0.0230919466 | 0.0221416184 | 0.0226086526 | 0.0193362965 | 0.0193362965 | 0.0662435129 | 0.0594698691 |
| *psaC* |  | 0.0000000000 | 0.0000000000 | 0.0000000000 | 0.0123279002 | 0.0249658200 | 0.0249658200 | 0.0419767566 | 0.0419767566 | 0.0377053972 | 0.0873507055 | 0.0733454535 |
| *psaI* |  | 0.0686514753 | 0.0076028549 | 0.0076028549 | 0.0066494790 | 0.0152988664 | 0.0152844542 | 0.0143226676 | 0.0152844542 | 0.0162624427 | 0.0410332404 | 0.0431840255 |
| *psaJ* |  | 0.0000000000 | 0.0078043942 | 0.0078043942 | 0.0078043942 | 0.0156788123 | 0.0156788123 | 0.0156788123 | 0.0238096232 | 0.0156788123 | 0.1201306113 | 0.0486701716 |
| *psbA* |  | 0.0066350270 | 0.0028332370 | 0.0028332370 | 0.0028332370 | 0.0212290052 | 0.0153155574 | 0.0202513913 | 0.0171939187 | 0.0171939187 | 0.0371171164 | 0.0462929721 |
| *psbC* |  | 0.0364269711 | 0.0073002894 | 0.0068399688 | 0.0068399688 | 0.0221416184 | 0.0230919466 | 0.0226086526 | 0.0193362965 | 0.0193362965 | 0.0662435129 | 0.0594698691 |
| *psbD* |  | 0.0000000000 | 0.0000000000 | 0.0000000000 | 0.0000000000 | 0.0179363786 | 0.0269600060 | 0.0179363786 | 0.0362934559 | 0.0362934559 | 0.0848609268 | 0.0551718990 |
| *psbE* |  | 0.0096953920 | 0.0000000000 | 0.0000000000 | 0.0000000000 | 0.0018825274 | 0.0037929290 | 0.0018825274 | 0.0018825274 | 0.0018825274 | 0.0199886284 | 0.0178267095 |
| *psbF* |  | 0.0000000000 | 0.0000000000 | 0.0000000000 | 0.0000000000 | 0.0084429156 | 0.0169421728 | 0.0084429156 | 0.0169421728 | 0.0169421728 | 0.0255141322 | 0.0254964735 |
| *psbH* |  | 0.0000000000 | 0.0045234025 | 0.0045234025 | 0.0045234025 | 0.0566769671 | 0.0566769671 | 0.0566769671 | 0.0614306594 | 0.0614306594 | 0.1327274520 | 0.1011685062 |
| *psbJ* |  | 0.0000000000 | 0.0081680405 | 0.0081680405 | 0.0081680405 | 0.0251388091 | 0.0251388091 | 0.0251388091 | 0.0251388091 | 0.0251388091 | 0.1020793094 | 0.0916901703 |
| *psbK* |  | 0.0108786588 | 0.0163548108 | 0.0163548108 | 0.0163548108 | 0.0394512627 | 0.0337378408 | 0.0394512627 | 0.0396432086 | 0.0396432086 | 0.1048173826 | 0.1158205914 |
| *psbM* |  | 0.0000000000 | 0.0000000000 | 0.0000000000 | 0.0000000000 | 0.0000000000 | 0.0096336877 | 0.0000000000 | 0.0193415511 | 0.0193415511 | 0.1477415046 | 0.0923474247 |
| *psbN* |  | 0.0000000000 | 0.0000000000 | 0.0000000000 | 0.0000000000 | 0.0076073074 | 0.0076073074 | 0.0076073074 | 0.0076073074 | 0.0076073074 | 0.0393924863 | 0.0154053906 |
| *psbT* |  | 0.0000000000 | 0.0000000000 | 0.0000000000 | 0.0000000000 | 0.0093631595 | 0.0093631595 | 0.0093631595 | 0.0000000000 | 0.0000000000 | 0.0580838482 | 0.0708245286 |
| *rbcL* |  | 0.0447899021 | 0.0070461483 | 0.0070461483 | 0.0070461483 | 0.0257205095 | 0.0257566436 | 0.0257205095 | 0.0206466830 | 0.0217062663 | 0.0654684483 | 0.0623341791 |
| *rpl2* |  | 0.0076603901 | 0.0000000000 | 0.0000000000 | 0.0000000000 | 0.0034174415 | 0.0030366926 | 0.0034174415 | 0.0022745431 | 0.0022745431 | 0.0296416096 | 0.0266625315 |
| *rpl14* |  | 0.0000000000 | 0.0164831117 | 0.0164831117 | 0.0164831117 | 0.0248806082 | 0.0221276974 | 0.0248806082 | 0.0306191407 | 0.0306191407 | 0.1189956602 | 0.0928410923 |
| *rpl16* |  | 0.0000000000 | 0.0077505569 | 0.0077505569 | 0.0077505569 | 0.0130024364 | 0.0129994831 | 0.0130024364 | 0.0090561362 | 0.0090561362 | 0.0441091359 | 0.0353210334 |
| *rpl20* |  | 0.0000000000 | 0.0390133019 | 0.0390133019 | 0.0390133019 | 0.0793912046 | 0.0829742252 | 0.0793912046 | 0.0760265671 | 0.0760265671 | 0.2303006895 | 0.2156839191 |
| *rpl22* |  | 0.0000000000 | 0.0085909628 | 0.0103205794 | 0.0085909628 | 0.0296098705 | 0.0234514562 | 0.0296098705 | 0.0260416139 | 0.0260416139 | 0.2303066955 | 0.3365008170 |
| *rpl23* |  | 0.0106265830 | 0.0000000000 | 0.0000000000 | 0.0000000000 | 0.0177614790 | 0.0177614790 | 0.0185523055 | 0.0141889399 | 0.0141889399 | 0.1115096339 | 0.0964558807 |
| *rpl32* |  | 0.0328269209 | 0.0000000000 | 0.0000000000 | 0.0000000000 | 0.0266481615 | 0.0266481615 | 0.0266481615 | 0.0334173399 | 0.0334173399 | 0.1329658055 | 0.1421104004 |
| *rpl33* |  | 0.0000000000 | 0.0203641315 | 0.0203641315 | 0.0203641315 | 0.0479332253 | 0.0479332253 | 0.0479332253 | 0.0535880272 | 0.0535880272 | 0.1825962752 | 0.1702321115 |
| *rpl36* |  | 0.0000000000 | 0.0088294154 | 0.0088294154 | 0.0088294154 | 0.0271143217 | 0.0271143217 | 0.0271143217 | 0.0179570223 | 0.0179570223 | 0.0764282488 | 0.1072422484 |
| *rpoA* |  | 0.0283935258 | 0.0170791167 | 0.0167564516 | 0.0167564516 | 0.0352305803 | 0.0342447085 | 0.0349043008 | 0.0303652607 | 0.0303652607 | 0.1029881573 | 0.0859746100 |
| *rpoB* |  | 0.0283935258 | 0.0170791167 | 0.0167564516 | 0.0167564516 | 0.0352305803 | 0.0342447085 | 0.0349043008 | 0.0303652607 | 0.0303652607 | 0.1029881573 | 0.0859746100 |
| *rpoC1* |  | 0.0374808634 | 0.0230490332 | 0.0232419193 | 0.0228760318 | 0.0464798802 | 0.0456684615 | 0.0460687129 | 0.0450668685 | 0.0449453454 | 0.1315180777 | 0.1089695462 |
| *rpoC2* |  | 0.0348415863 | 0.0175564780 | 0.0175564780 | 0.0175564780 | 0.0425213265 | 0.0437068058 | 0.0425213265 | 0.0425387109 | 0.0422720063 | 0.1478660871 | 0.1238921575 |
| *rps2* |  | 0.0868961094 | 0.1116215778 | 0.1116215778 | 0.1099014949 | 0.1099014949 | 0.1099014949 | 0.1015552085 | 0.1015552085 | 0.1080680525 | 0.1080680525 | 0.1080680525 |
| *rps3* |  | 0.0018376198 | 0.0132278108 | 0.0132278108 | 0.0132278108 | 0.0230650930 | 0.0220820041 | 0.0230650930 | 0.0230242610 | 0.0230242610 | 0.0689107973 | 0.0553431775 |
| *rps4* |  | 0.0000000000 | 0.0201367940 | 0.0201367940 | 0.0201367940 | 0.0355689837 | 0.0355689837 | 0.0355689837 | 0.0391125717 | 0.0391125717 | 0.1045338825 | 0.0923242494 |
| *rps7* |  | 0.0557806825 | 0.0000000000 | 0.0000000000 | 0.0000000000 | 0.0396789994 | 0.0396789994 | 0.0396789994 | 0.0419737426 | 0.0419737426 | 0.0603891240 | 0.0509681687 |
| *rps8* |  | 0.0000000000 | 0.0139056883 | 0.0139056883 | 0.0139056883 | 0.0187472536 | 0.0187472536 | 0.0155587505 | 0.0155587505 | 0.0155587505 | 0.0874210939 | 0.0755788294 |
| *rps11* |  | 0.0446023534 | 0.0813908100 | 0.0813908100 | 0.0813908100 | 0.1060885620 | 0.1060940203 | 0.1060885620 | 0.1063064100 | 0.1063064100 | 0.1580748291 | 0.1373432149 |
| *rps12* |  |  |  |  |  |  |  |  |  |  |  |  |
| *rps14* |  | 0.0086218274 | 0.0134850502 | 0.0134850502 | 0.0134850502 | 0.0342589213 | 0.0306555497 | 0.0342589213 | 0.0377734263 | 0.0377734263 | 0.1155825338 | 0.0997877126 |
| *rps15* |  | 0.0146801188 | 0.0000000000 | 0.0000000000 | 0.0000000000 | 0.0222001471 | 0.0262914342 | 0.0222001471 | 0.0334878304 | 0.0334878304 | 0.1421478992 | 0.1591637175 |
| *rps18* |  | 0.0476068940 | 0.0363634501 | 0.0363634501 | 0.0363634501 | 0.0477614934 | 0.0477614934 | 0.0477614934 | 0.0439358538 | 0.0439358538 | 0.1368325333 | 0.1152954454 |

**Table S5.** Codon Usage in *T. hamiensis* var. *qatarensis* and *T. simplex* plastomes

| Codon | Amino acid | Frequency | | Number | |
| --- | --- | --- | --- | --- | --- |
|  |  | TQ | TS | TQ | TS |
| GCA | A | 18.338 | 17.627 | 369 | 480 |
| GCC | A | 7.902 | 8.373 | 159 | 228 |
| GCG | A | 5.019 | 6.023 | 101 | 164 |
| GCT | A | 32.8 | 27.946 | 660 | 761 |
| TGC | C | 4.324 | 3.709 | 87 | 101 |
| TGT | C | 10.436 | 8.85 | 210 | 241 |
| GAC | D | 8.051 | 7.895 | 162 | 215 |
| GAT | D | 24.848 | 27.799 | 500 | 757 |
| GAA | E | 37.173 | 37.714 | 748 | 1027 |
| GAG | E | 10.635 | 11.127 | 214 | 303 |
| TTC | F | 17.742 | 18.876 | 357 | 514 |
| TTT | F | 39.062 | 39.11 | 786 | 1065 |
| GGA | G | 25.892 | 28.24 | 521 | 769 |
| GGC | G | 6.759 | 7.308 | 136 | 199 |
| GGG | G | 11.579 | 12.412 | 233 | 338 |
| GGT | G | 29.073 | 25.779 | 585 | 702 |
| CAC | H | 4.87 | 6.169 | 98 | 168 |
| CAT | H | 19.034 | 20.198 | 383 | 550 |
| ATA | I | 25.594 | 25.008 | 515 | 681 |
| ATC | I | 13.766 | 13.991 | 277 | 381 |
| ATT | I | 43.932 | 41.937 | 884 | 1142 |
| AAA | K | 39.956 | 41.937 | 804 | 1142 |
| AAG | K | 12.275 | 13 | 247 | 354 |
| CTA | L | 11.878 | 11.972 | 239 | 326 |
| CTC | L | 5.318 | 6.28 | 107 | 171 |
| CTG | L | 5.019 | 5.545 | 101 | 151 |
| CTT | L | 21.37 | 20.895 | 430 | 569 |
| TTA | L | 35.881 | 34.813 | 722 | 948 |
| TTG | L | 19.63 | 20.381 | 395 | 555 |
| ATG | M | 22.811 | 21.997 | 459 | 599 |
| AAC | N | 11.033 | 11.311 | 222 | 308 |
| AAT | N | 31.061 | 32.61 | 625 | 888 |
| CCA | P | 12.076 | 12.119 | 243 | 330 |
| CCC | P | 6.262 | 6.316 | 126 | 172 |
| CCG | P | 4.92 | 5.876 | 99 | 160 |
| CCT | P | 17.394 | 15.13 | 350 | 412 |
| CAA | Q | 24.053 | 25.339 | 484 | 690 |
| CAG | Q | 8.299 | 9.217 | 167 | 251 |
| AGA | R | 18.736 | 19.279 | 377 | 525 |
| AGG | R | 6.759 | 6.61 | 136 | 180 |
| CGA | R | 12.275 | 12.192 | 247 | 332 |
| CGC | R | 3.727 | 4.223 | 75 | 115 |
| CGG | R | 3.777 | 3.415 | 76 | 93 |
| CGT | R | 15.108 | 13.147 | 304 | 358 |
| AGC | S | 4.97 | 6.83 | 100 | 186 |
| AGT | S | 13.816 | 14.689 | 278 | 400 |
| TCA | S | 13.07 | 13.551 | 263 | 369 |
| TCC | S | 9.84 | 10.429 | 198 | 284 |
| TCG | S | 3.578 | 5.912 | 72 | 161 |
| TCT | S | 22.711 | 21.116 | 457 | 575 |
| ACA | T | 16.4 | 16.305 | 330 | 444 |
| ACC | T | 9.393 | 9.291 | 189 | 253 |
| ACG | T | 4.025 | 4.04 | 81 | 110 |
| ACT | T | 25.793 | 21.63 | 519 | 589 |
| GTA | V | 23.705 | 22.989 | 477 | 626 |
| GTC | V | 6.162 | 6.61 | 124 | 180 |
| GTG | V | 7.057 | 7.455 | 142 | 203 |
| GTT | V | 21.817 | 20.014 | 439 | 545 |
| TGG | W | 21.916 | 20.602 | 441 | 561 |
| TAC | Y | 7.256 | 7.381 | 146 | 201 |
| TAT | Y | 29.967 | 29.268 | 603 | 797 |
| TAA | * | 5.765 | 5.435 | 116 | 148 |
| TAG | * | 2.833 | 2.975 | 57 | 81 |
| TGA | * | 3.479 | 3.782 | 70 | 103 |

TQ: *T. qatarensis*, TS: *T. simplex*
